# The biogenesis and function of nucleosome arrays

**DOI:** 10.1038/s41467-021-27285-6

**Published:** 2021-12-01

**Authors:** Ashish Kumar Singh, Tamás Schauer, Lena Pfaller, Tobias Straub, Felix Mueller-Planitz

**Affiliations:** 1 Molecular Biology, Biomedical Center, Faculty of Medicine, Ludwig-Maximilians-Universität München, 82152 Planegg-Martinsried, München, Germany; 2 Bioinformatics Unit, Biomedical Center, Faculty of Medicine, Ludwig-Maximilians-Universität München, 82152 Planegg-Martinsried, München, Germany; 3 Institute of Physiological Chemistry, Faculty of Medicine Carl Gustav Carus, Technische Universität Dresden, Fetscherstraße 74, 01307 Dresden, Germany; 4 Present Address: Novartis Institutes for BioMedical Research, 4056 Basel, Switzerland

**Keywords:** DNA, Chromatin, Epigenetics

## Abstract

Numerous chromatin remodeling enzymes position nucleosomes in eukaryotic cells. Aside from these factors, transcription, DNA sequence, and statistical positioning of nucleosomes also shape the nucleosome landscape. The precise contributions of these processes remain unclear due to their functional redundancy in vivo. By incisive genome engineering, we radically decreased their redundancy in *Saccharomyces cerevisiae*. The transcriptional machinery strongly disrupts evenly spaced nucleosomes. Proper nucleosome density and DNA sequence are critical for their biogenesis. The INO80 remodeling complex helps space nucleosomes in vivo and positions the first nucleosome over genes in an H2A.Z-independent fashion. INO80 requires its Arp8 subunit but unexpectedly not the Nhp10 module for spacing. Cells with irregularly spaced nucleosomes suffer from genotoxic stress including DNA damage, recombination and transpositions. We derive a model of the biogenesis of the nucleosome landscape and suggest that it evolved not only to regulate but also to protect the genome.

## Abstract (web summary)

Nucleosomes form arrays with even spacing between them in virtually all eukaryotes; however, their biogenesis is incompletely understood. Here the authors show that nucleosome density and DNA sequence along with the Ino80 chromatin remodeling complex play a role in nucleosome array formation in yeast and that the transcriptional machinery disrupts evenly-spaced nucleosomes.

## Introduction

Nucleosomes are an ancient innovation of evolution and shape the structure and function of genomes of virtually all eukaryotes. DNA is densely coated with these particles, which profoundly influences access to the underlying genetic information and affects all nuclear processes^1^.

Nucleosomes arrange on DNA like beads on a string, forming arrays of evenly spaced nucleosomes with a characteristic center-to-center distance, the so-called nucleosome repeat length (NRL). Nucleosome arrays tend to be aligned (“phased”), with respect to the transcription start site (TSS). They are punctuated by nucleosome free regions (NFRs) around promoters. NFRs are important for promoter activity^2^, and the position of the first nucleosome downstream of the TSS, the +1 nucleosome, helps select the precise start site of transcription^3,4^.

The nucleosome landscape is under constant assault from disruptive processes such as DNA replication and repair^5^. ATP-dependent nucleosome remodelers are important factors that help to reestablish the nucleosome landscape^6,7^. The RSC remodeler, for instance, clears the NFR of nucleosomes and thereby contributes to proper positioning of the +1 nucleosome^8^. Other remodelers specialize in generating equal spacing between nucleosomes. This activity is well documented for ISWI and Chd1 remodelers. Cells lacking these “spacing remodelers” suffer from disrupted nucleosome arrays^9–11^ and closely packed di-nucleosomes.

How spacing remodelers work mechanistically remains contested. Evidence for two scenarios exists. One model posits that spacing remodelers set a characteristic NRL between nucleosomes by “clamping” nucleosomes at fixed distances^12,13^. The second model proposes that the remodeler measures the length of DNA that flanks the nucleosome, the so-called linker DNA. Long linker DNA activates the remodeler, which then slides the nucleosome efficiently in the direction of the long linker. In doing so, it equilibrates the linker lengths in an array of nucleosomes^14^.

Nucleosome spacing is difficult to assay in vitro due to a dearth of sensitive spacing assays. The movement of a mononucleosome to the center of a short DNA molecule often serves as a proxy of the spacing reaction. ISWI, Chd1, and INO80 are all able to center mononucleosomes on DNA^15,16^. Consistent with the linker length equilibration model, all these remodelers sensitively react to the length of DNA flanking the nucleosome. INO80, for example, slides nucleosomes ~100-fold faster when the flanking DNA length increases from 40 to 60 bp. Responsible for this switch-like response is its Nhp10 module, which binds to flanking DNA^17^. The Arp8 module also binds linker DNA and, like Nhp10, contributes to linker DNA sensing^18,19^. Linker length sensing through these subunits may underlie INO80’s ability to position trinucleosomes with ~30 bp distances^16^.

In principle, nucleosome arrays could emerge even without the help of remodelers through statistical positioning^20^. This possibility requires a nucleosome-repellent barrier, which DNA-binding proteins, such as General Regulatory Factors (GRFs), or certain DNA sequences are known to form^2^. When the nucleosome density is high, nucleosomes downstream of the barrier can only assume a limited number of configurations. After population averaging, the limited number of configurations leads to what looks like evenly spaced nucleosome arrays, even though nucleosomes may not be evenly spaced on individual DNA molecules.

A critical test of statistical positioning is reducing the number of nucleosomes. Average distances between nucleosomes should increase, and nucleosome positioning should decrease in the population average. Results from nucleosome depletion experiments in yeast, however, were not fully conclusive. Whereas nucleosome depletion decreased the positioning of nucleosomes, spacing between nucleosomes did not seem to increase sufficiently^21–23^. Perhaps nucleosomes remain clamped together by remodeling enzymes^12,13^.

In addition to remodelers and statistical positioning, the transcription machinery has been suggested to play pivotal roles in the biogenesis of the nucleosome architecture. The RNA polymerase II preinitiation complex, in conjunction with other factors, could help generate the NFR and position the +1 nucleosome. The +1 nucleosome would then serve as a barrier against which other nucleosomes are packed. In addition, transcription elongation may directly or indirectly establish nucleosome arrays, e.g., through recruiting spacing remodelers^24,25^. In vitro experiments also hint at a role of transcription in organizing the nucleosome landscape. Transcriptionally inactive cell extracts qualitatively establish features of in vivo like nucleosome patterns on in vitro reconstituted nucleosomes, but not to the degree seen in vivo^26^.

The large number of processes acting on the nucleosome landscape has hampered previous efforts to disentangle their contributions and study their mechanisms, limiting our understanding of the biogenesis of the nucleosome landscape. Here, we exploit a yeast strain lacking all remodelers of the ISWI and Chd1 family (*isw1*Δ, *isw2*Δ, *chd1*Δ; referred to as TKO hereafter)^27^ to reduce functional redundancy. This strategy helped us to reconcile contradictory interpretations with regards to contributions of DNA sequence, statistical positioning and transcription towards the biogenesis of the nucleosome landscape. We present evidence that INO80 not only positions +1 nucleosomes. It can also space nucleosomes over the majority of genes in a transcription-independent fashion in vivo. INO80 does so by relying on the Arp8 but not the Nhp10 subunit or the histone variant H2A.Z. Finally, transposition, recombination and DNA damage assays support the notion that even spacing between nucleosomes protect the genome from genotoxic insults. Overall, our results suggest that transcription and nucleosome remodelers compete to establish nucleosome arrays, which helps maintain genome integrity.

## Results

### Nucleosome density and remodelers cooperate to generate nucleosome arrays

Nucleosome arrays are thought to persist after artificial reduction of histones in vivo. They appear to possess NRLs that are similar to those of WT cells^21–23^. We hypothesized that these residual arrays are the product of spacing remodelers of the ISWI and CHD1 family, which we found before to clamp nucleosomes at fixed distances in vitro^12^. To test the clamping hypothesis in vivo, we employed a histone depletion (HD) system^28^ in cells lacking *ISW1*, *ISW2*, and *CHD1* (TKO HD). Histone levels were approximately halved compared to WT cells (Supplementary Fig. 1a, b).

**Fig. 1:**
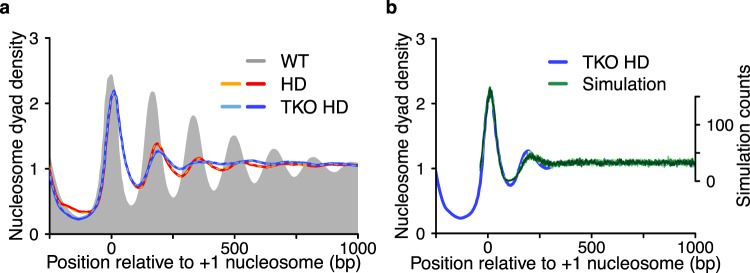
ISWI and Chd1 remodelers rely on proper nucleosome densities to generate nucleosome arrays. **a** Composite plots depicting the average nucleosome organization of ~5000 genes before (WT; grey) and after histone depletion in otherwise wild-type (HD) or TKO cells (TKO HD). All strains express histones H3 and H4 from a galactose promoter. HD was induced by shifting cells from galactose-containing to glucose-containing media for 3 h, which reduced histone protein amounts by ~50% (Supplementary Fig. 1a, b). Nucleosome dyad positions were aligned to known +1 nucleosome positions of WT cells. Dashed lines in brighter color are biological replicates. **b** Simulated, truly random nucleosome organization downstream of a well-positioned +1 nucleosome. Nine simulations are overlayed (green). Nucleosome occupancy was fixed at 51% to simulate histone depletion conditions (see “Methods” section). TKO HD data from **a** is replotted for reference.

Composite plots reveal that the +1 nucleosomes remain well positioned in the TKO HD strain as suggested by a largely comparable peak height of the +1 in WT and TKO HD samples (Fig. 1a and Supplementary Fig. 1c). The +2 nucleosomes are still discernible in TKO HD. Beyond the +2 nucleosomes, however, phased and evenly spaced nucleosome arrays (hereafter simply referred to as regular nucleosome arrays) are largely absent.

We wondered if the +2 nucleosomes in the TKO HD cells are actively positioned by unknown mechanisms or if their positioning emerges from random, i.e., statistical positioning. To test the latter possibility, we simulated random nucleosome positions downstream of a well-positioned +1 nucleosome at a nucleosome density that mimics in vivo histone depletion (see “Methods” section). The simulated curve showed a +2 nucleosome at a similar location to the TKO HD sample (Fig. 1b), suggesting that the +2 nucleosomes in the TKO HD sample emerge at least to some extent from statistical positioning.

To test whether spacing remodelers of the ISWI and Chd1 family can impose order in the randomly positioned nucleosomes present in TKO HD cells, we performed MNase-seq in histone-depleted but otherwise WT cells. Compared to TKO HD, nucleosome arrays in HD cells experienced a modest but consistent increase in amplitude (Fig. 1a). This increase is not caused by changes in cell cycle progression as both HD and TKO HD cells enrich to a similar extent in the G2/M phase (Supplementary Fig. 1d)^29^.

The results imply that ISWI and/or Chd1 remodelers can order nucleosomes after histone depletion, consistent with a clamping activity^12^. However, amplitudes for nucleosome arrays in HD cells only modestly exceeded those of TKO HD. Moreover, they fell drastically short of those in WT cells (Fig. 1a). We, therefore, propose that ISWI and Chd1 remodelers cannot overcome the disorder induced by reducing the nucleosome density. High histone density is thus required by ISWI and Chd1 to generate nucleosome arrays.

### Transcription destroys the nucleosome landscape

**Fig. 2:**
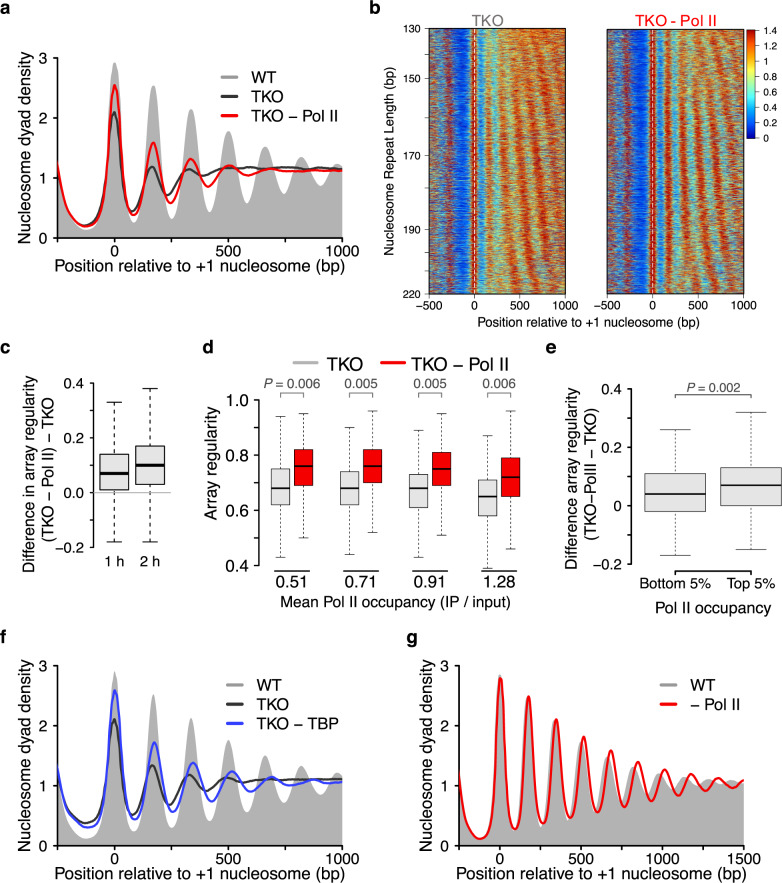
Transcription disrupts the regularity of nucleosome arrays. **a** Pol II depletion in TKO cells using anchor-away technology elevates levels of regular nucleosome arrays (red) compared to a TKO control strain (black). A WT control strain is shown for reference (grey). Rpb1 is FRB-tagged in all strains. Cells were either rapamycin-treated or mock-treated with vehicle for 1 h. **b** Heatmaps for data from **a**. Genes were sorted according to the NRL observed in TKO cells. Color scale represents nucleosome dyad density. **c** Most genes acquire a higher degree of array regularity after Pol II depletion in TKO cells. Shown is the difference in array regularity for each gene before and after Pol II depletion for 1 and 2 h. Boxplot represent array regularity calculated on pooled data (three replicates for 1 h and one replicate for 2 h depletion). **d** Array regularity is rescued upon Pol II depletion irrespective of the transcriptional strength of genes. Genes were sorted by Pol II occupancy and divided into quartiles. Mean Pol II occupancy values of each quartile can be found underneath. Pol II occupancy data for an *isw1*Δ, *chd1*Δ double mutant strain served as a proxy for TKO cells^10^. Similar results were obtained when genes were sorted by gene expression^56^. Statistical analysis represents linear mixed-effect model fitted on mean array regularity values of two replicates. **e** Change in array regularity upon Pol II depletion in TKO cells in genes with top and bottom 5% Pol II occupancy. *P*-value (*P*) represent statistical analyses performed with two-tailed Welch’s *t*-test on the mean values of three replicates. **f** Same as **a**, but for TBP depletion (1 h) in TKO cells. **g** Nucleosome organization upon Pol II depletion (1 h) in WT cells. Central lines in box plots indicate the median, the box shows the interquartile range, and whiskers indicate data points within 1.5× of the interquartile range.

Albeit the simulation was qualitatively similar to TKO HD data, the +2 peak in TKO HD data appeared to be sharper than in the simulation. Additional factors, e.g., the transcriptional machinery, could be responsible for this residual nucleosome organization^7,24,25^. Consistent with this notion, TKO cells with normal histone levels also possess detectable arrays even though the cells are devoid of bona fide spacing remodelers (Fig. 2a, b)^9,11^.

If transcription contributed to array formation, one would expect a decline in nucleosome array organization upon transcription inhibition. To test this possibility, we depleted the largest subunit of Pol II (Rpb1) for 1 and 2 h in TKO using anchor-away technology (Supplementary Fig. 2a)^30^. Contrary to the expectation, nucleosome arrays became more pronounced when we blocked transcription (Fig. 2a and Supplementary Fig. 2b–d). To test whether only a subset of genes experiences a rescue of nucleosome arrays upon Pol II depletion, we measured the NRL^10^ and array regularity^31^ for each gene (Supplementary Fig. 2e). Heatmaps of the NRL-sorted MNase-seq data reveal that Pol II depletion globally affects the nucleosome landscape and NRL distribution (Fig. 2b and Supplementary Fig. 2f). Average array regularity reproducibly increased over most genes after Pol II depletion. The majority of the effect was obtained after 1 h depletion already (Fig. 2c and Supplementary Fig. 2g; 77.5% for 1 h and 81% for 2 h). The increase in array regularity upon Pol II depletion affected the entire genome, also lowly transcribed genes (Fig. 2d), consistent with the pervasive transcription of the genome^32^. Compared to highly transcribed genes, lowly transcribed genes possessed higher array regularity (Supplementary Fig. 2h) and experienced a lower rescue of regularity upon Pol II depletion (Fig. 2e). Overall, these results suggest a direct role of transcription in destroying array regularity.

Pol II depletion shifts cells towards the G1 phase (Supplementary Fig. 2i) and could therefore increase array regularity indirectly. We ruled out this possibility by depleting Pol II in fully arrested TKO cells (Supplementary Fig. 2j, k). Rapamycin treatment of the TKO control strain lacking an FRB-tag on Pol II (Supplementary Data 1) did not affect nucleosome organization either because it remains similar to vehicle-treated TKO cells containing the FRB tag. Other indirect effects remain possible. However, given that we observe a gain of regularity, not a destruction, and that we have identified the factor that causes the increase (see below), we consider indirect effects less likely.

The results above leave open the possibility that components of the transcription pre-initiation complex (PIC) can still assemble after Pol II depletion and serve as a barrier for phasing arrays^33^. To test this possibility, we depleted the TATA-binding protein (TBP) in TKO cells (Supplementary Fig. 2l). TBP depletion rescued nucleosome arrays to a similar extent as Pol II depletion (Fig. 2f), suggesting that the PIC does not contribute towards establishing regular nucleosome arrays.

The results so far do not support models in which the transcription machinery promotes nucleosome array organization. A caveat remains, however, as transcription may promote array formation only in presence of all remodelers, for instance by recruiting them to chromatin. According to this hypothesis, Pol II depletion in WT cells should decrease regular nucleosome arrays. However, array regularity was not compromised upon depletion of Pol II in WT cells (Fig. 2g and Supplementary Fig 2a). In fact, in six available datasets collected in three laboratories, array regularity increased, not decreased, after Pol II depletion (Supplementary Fig 3b–e)^34,35^. We, therefore, suggest that the act of transcription is disruptive to nucleosome arrays even in presence of spacing remodelers.

### The INO80 remodeling complex induces spacing of nucleosomes in vivo

The rescue of array regularity upon Pol II depletion in TKO cells was intriguing because it implies the existence of another spacing factor in addition to the bona fide spacers of the ISWI and Chd1 family. The INO80 complex is an attractive candidate because its deletion in WT cells shifts genic nucleosomes towards TSS^23,36^ and it can equalize linker lengths in trinucleosomes in vitro^16,26^.

**Fig. 3:**
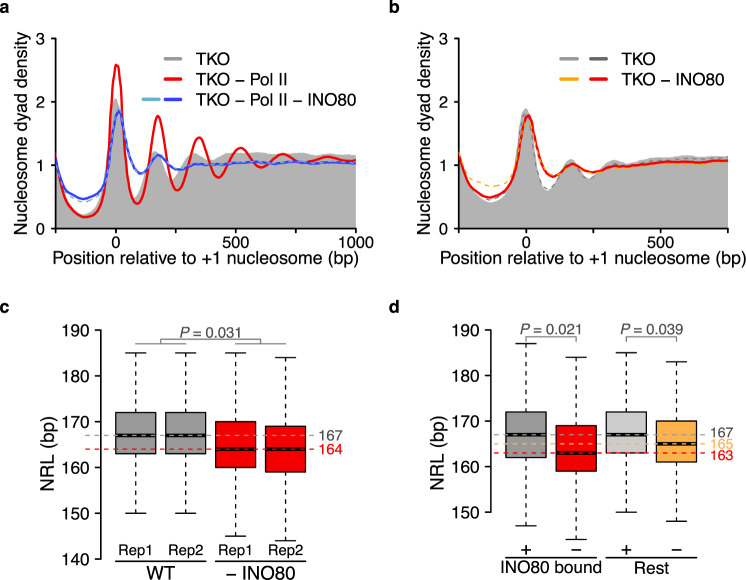
INO80 induces nucleosome array formation in vivo. **a** Nucleosome organization for the indicated Pol II anchor-away strains. Samples were treated with vehicle (TKO) or rapamycin (all other samples) for 2 h, twice as long as in Fig. 2. The treatment regimen allowed for efficient depletion, as tested by live cell imaging of GFP-tagged INO80 and Rbp3 ChIP-qPCR (Supplementary Figs. 2a and 4a). Dashed line: biological replicate. **b** Nucleosome organization upon INO80 depletion (1.5 h) in TKO cells (red and orange). Ino80-GFP-FRB tagged TKO cells treated with vehicle served as the reference (grey). Live cell imaging confirmed INO80 depletion (Supplementary Fig. 4d). **c** NRL distribution before and after INO80 depletion (1.5 h) in WT cells. Median values are indicated with horizontal dashed lines. **d** NRLs of 1646 genes bound by INO80, as measured by ChEC-seq^3^. Bound genes have median value (red dashed line) that is 2 bp shorter of that of all other genes (orange dashed line) after depletion. *P*-values (*P*) in **c**, **d** represent statistical analyses performed with two-tailed Welch’s *t*-test on the mean values of two replicates. Central lines in box plots indicate the median, the box shows the interquartile range, and whiskers indicate data points within 1.5 times of the interquartile range.

To test whether the INO80 complex is responsible for the rescue upon Pol II depletion, we co-depleted INO80 and Pol II in TKO cells (Supplementary Figs. 2a and 4a). Unlike Pol II single depletion, co-depletion of Pol II and INO80 did not increase the regularity of nucleosome arrays (Fig. 3a and Supplementary Fig. 4b, c). The co-depletion result is consistent with INO80 being the responsible factor that forms arrays after Pol II depletion, albeit indirect effects occurring during co-depletion cannot be ruled out. Supporting the notion that INO80 helps establish regular nucleosome arrays, its depletion in actively transcribing TKO cells decreased nucleosome array regularity (Fig. 3b and Supplementary Fig. 4d, e). We ruled out that these effects were confined to genes that suffer from an altered Pol II distribution upon INO80 depletion (Supplementary Fig. 4f, g). The combined results imply that INO80 helps generate phased, regular nucleosome arrays genome-wide in TKO cells, and that it can do so in the presence or absence of active transcription.

We next tested if INO80 contributes to the nucleosome landscape also in WT cells. Its depletion from WT cells consistently reduced the median NRL by 3 bp in two biological replicates. The same was true when we reanalyzed published data^37^ (Fig. 3c and Supplementary Fig. 4j). INO80-bound genes^3^ experienced a larger decrease (4 bp) compared to all other genes (2 bp) (Fig. 3d). An alteration in cell cycle progression upon INO80 depletion^38^ is unlikely to cause NRL contraction because the NRL distribution does not change during the cell cycle (Supplementary Fig. 4k)^39^. Depletion of INO80 also diminished nucleosome regularity, albeit to varying degrees in our and published data (Supplementary Fig. 4h, i). Overall, these results suggest that INO80 contributes to NRL determination in WT cells.

### The Arp8, not the Nhp10 module regulates INO80 spacing in vivo

**Fig. 4:**
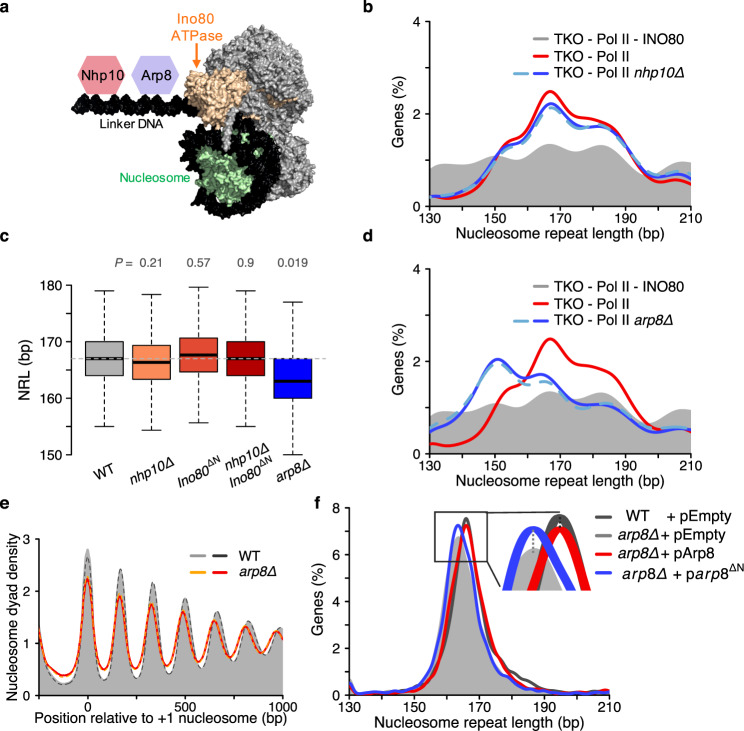
Arp8, but not Nhp10, regulates INO80-mediated spacing in vivo. **a** Cartoon of the INO80 complex bound to the nucleosome based on PDB code 6FML https://www.rcsb.org/structure/6fml. The Nhp10 and Arp8 modules interact with linker DNA. **b** Deletion of *NHP10* (blue lines) does not substantially alter the NRL distribution in TKO-Pol II samples (red; all colored lines peak at 167 bp). Cells depleted for INO80 are shown as a reference (grey). **c** Box plots of measured NRLs in the indicated single and double mutants in otherwise WT cells. Horizontal line is at 167 bp. *P* values are from a two-tailed paired Welch’s t-test on the mean values of two replicates compared to WT. **d** Same as **b**, but for *arp8Δ*. The NRL distribution shifts left peaking at 151 bp. **e** *ARP8* deletion affects the amplitude and NRL of arrays. **f** *ARP8* deletion shifts the NRL distribution to shorter values (peaks at 163 bp for *arp8Δ* and 166 bp for WT cells). This effect can be faithfully rescued by expression of wild-type Arp8 but not Arp8 lacking amino acids 2–197 (dashed lines). pEmpty: empty vector. Central line in box plot indicates the median, the box shows the interquartile range, and whiskers indicate data points within 1.5 times of the interquartile range.

Our ability to isolate INO80 activity provided us with the opportunity to dissect the spacing mechanism of INO80 in vivo (Fig. 4a). The Nhp10 module of INO80 imparts a switch-like response on the sliding activity in response to small changes in the linker length^17^. As such, the Nhp10 module is a strong candidate to control nucleosome spacing. To test this possibility in vivo, we deleted *NHP10* from the TKO-Pol II system. While the amplitude of arrays modestly reduced upon *NHP10* deletion (Supplementary Fig. 5a), the NRL distribution with and without Nhp10 remained similar (Fig. 4b). Comparable effects were observed after *NHP10* deletion in transcribing WT and TKO cells (Fig. 4c and Supplementary Fig. 5b, d)^40^ and after deletion of the 300 N-terminal amino acid residues of the Ino80 ATPase subunit (Fig. 4c and Supplementary Fig. 5c, e). These residues contribute to the association of the Nhp10 module to INO80 and proteolytically degrade when Nhp10 is missing^17,41^ (Supplementary Fig. 5f). Concurrent deletion of *NHP10* and the N-terminus of Ino80 in WT and TKO cells also showed negligible effects (Fig. 4c). Collectively, the results challenge the hypothesis that the Nhp10 module or the N-terminus of Ino80 are critically required for the INO80 complex to evenly space nucleosomes and to determine INO80’s preferred genome-wide NRL.

Like Nhp10, Arp8 has been implicated in linker DNA sensing^18,19^. When we deleted *ARP8*, INO80 lost much of its ability to evenly space nucleosomes in Pol II-depleted TKO cells (Supplementary Fig. 5g), attesting to Arp8’s important catalytic role^42^. Nevertheless, the residual arrays generated in absence of Arp8 had an NRL distribution that was strongly shifted to shorter values, peaking at 151 bp instead of the 167 bp observed in presence of Arp8 (Fig. 4d and Supplementary Fig. 5h). A shift to smaller NRL upon *ARP8* deletion can be observed also in WT and TKO cells (Fig. 4e, f and Supplementary Fig. 5i, j). This shift could be faithfully rescued by expression of WT Arp8, but not Arp8 lacking 197 N-terminal amino acids, which interact with the linker DNA^18^ (Fig. 4f).

*ARP8* deletion does not fully phenocopy INO80 depletion, as suggested by the partial overlap of genes that experienced NRL changes upon removal of the factors (Supplementary Fig. 5k). Altered catalytic properties of Arp8-less INO80, such as changes in linker length sensing, likely contribute to this effect, and caution against using *arp8*Δ as a proxy for a full *INO80* deletion.

In conclusion, these results highlight the importance of Arp8 and its N-terminal region for INO80-mediated remodeling and suggest that Arp8 helps INO80 to determine the NRL.

### INO80 positions the +1 nucleosome independently of H2A.Z in vivo

**Fig. 5:**
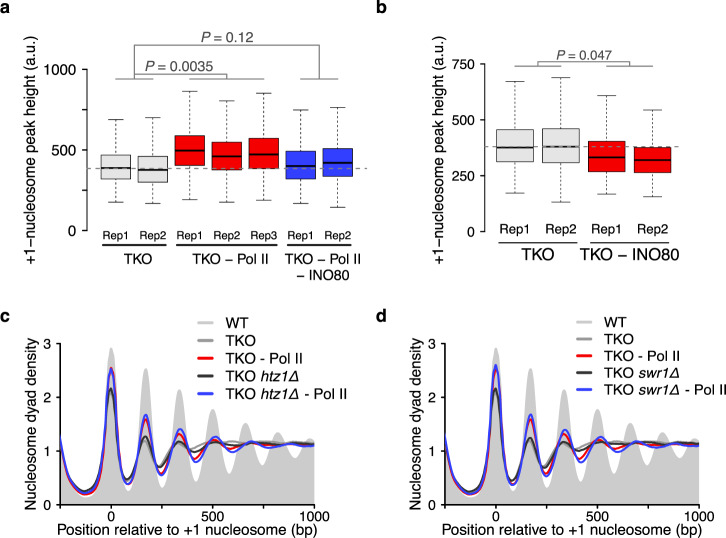
IN080 positions +1 nucleosomes independently of H2A.Z. **a** Box plots of peak heights measured for +1 nucleosomes of ∼5000 genes in the indicated strains and replicates. TKO is a Rpb1-FRB tagged strain treated with vehicle for 1 h. The horizontal dashed line indicates the mean of two biological replicates of the TKO strain. P-values (*P*) represent statistical analyses performed with one-way Anova followed by post-hoc Tukey HSD test on the mean values of individual replicates. **b** Same as **a**, but for INO80 depletion in TKO cells. TKO reference: Ino80-GFP-FRB tagged TKO cells treated with vehicle. Statistical analysis was performed with two-tailed paired Welch’s *t*-test on the mean values of two replicates. **c** Deletion of H2A.Z (*htz1Δ*; blue) has no deleterious effects on +1 peak heights and nucleosome arrays upon Pol II depletion (red). The negative controls (grey and black) are the corresponding vehicle-treated Rpb1-FRB tagged strain. Pol II was depleted for 1 h. **d** Same as **c**, but for *swr1*Δ. Data in **c** and **d** are averages of two or three biological replicates. Central lines in box plots indicate the median, the box shows the interquartile range, and whiskers indicate data points within 1.5 times of the interquartile range.

The results above indicate that INO80 helps increase the levels of phased regular arrays. It could do so by spacing nucleosomes in gene bodies and/or by positioning the +1 nucleosome. We noticed that the +1 peak was more pronounced after Pol II depletion in TKO cells in composite plots, and that this increase depended on the presence of INO80 (Fig. 3a). To substantiate this observation, we measured +1 nucleosome peak intensities at each gene^43^. Peak intensities of most +1 nucleosome increased after Pol II depletion in an INO80-dependent manner (Fig. 5a). Moreover, +1 peak intensities reduced upon INO80 depletion in TKO cells (Fig. 5b). We therefore suggest that the INO80 complex has a role in positioning the +1 nucleosome in vivo, in line with previous observations^26,37^.

The +1 nucleosome is strongly enriched with the histone variant H2A.Z, particularly upon Pol II depletion^34^. INO80 slides H2A.Z-containing nucleosomes two-fold to four-fold faster than canonical nucleosomes in vitro^44,45^, leading to a simple model that INO80 recognizes H2A.Z and thereby positions the +1 nucleosomes particularly well. Strikingly, H2A.Z deletion had no adverse effect on the peak height and width of the +1 nucleosome in TKO cells, even after Pol II depletion where INO80 activity can be visualized best (Fig. 5c). We independently validated these results by deleting the SWR1 remodeler, which deposits H2A.Z at +1 nucleosomes (Fig. 5d). We conclude that INO80 positions +1 nucleosomes independently of H2A.Z.

### The DNA sequence affects the NRL

The location of the +1 nucleosome is encoded in part by nucleosome-attracting DNA sequences^46^. Close inspection reveals that +1 nucleosomes tend to reside 17 bp downstream of the thermodynamically most preferred positions (Table 1). Depletion of RSC^47^ returns the +1 nucleosomes to within 1 bp of the nucleosome-attracting DNA sequences. Upon INO80 depletion, the +1 nucleosomes moved into the gene body, further away from the thermodynamically favored position (Table 1). Such an effect has been observed before and agrees with the suggestion that INO80 and RSC engage in a tug-of-war over the positions of the +1 nucleosomes^3^.

**Table 1:** Distances between MNase-Seq derived and calculated nucleosome affinities in the +1 nucleosome position.

Strain	Distance (bp)^a^
WT	17 ± 0(4)
−Pol II	17.5 ± 0.5(2)
−RSC^b^	1.33 ± 0.58(3)
TKO	17.5 ± 0.5(4)
TKO-INO80	27 ± 0(2)
TKO-Pol II	17.5 ± 0.5(4)
TKO-Pol II-INO80	29.5 ± 0.5(2)

^a^The +1 nucleosome position was determined from peak positions in gene-averaged nucleosome organization for MNase-Seq and nucleosome affinities^48^. Mean ± SD of replicates; values in brackets are number of replicates.

^b^MNase-Seq data from ref. ^47^.

**Fig. 6:**
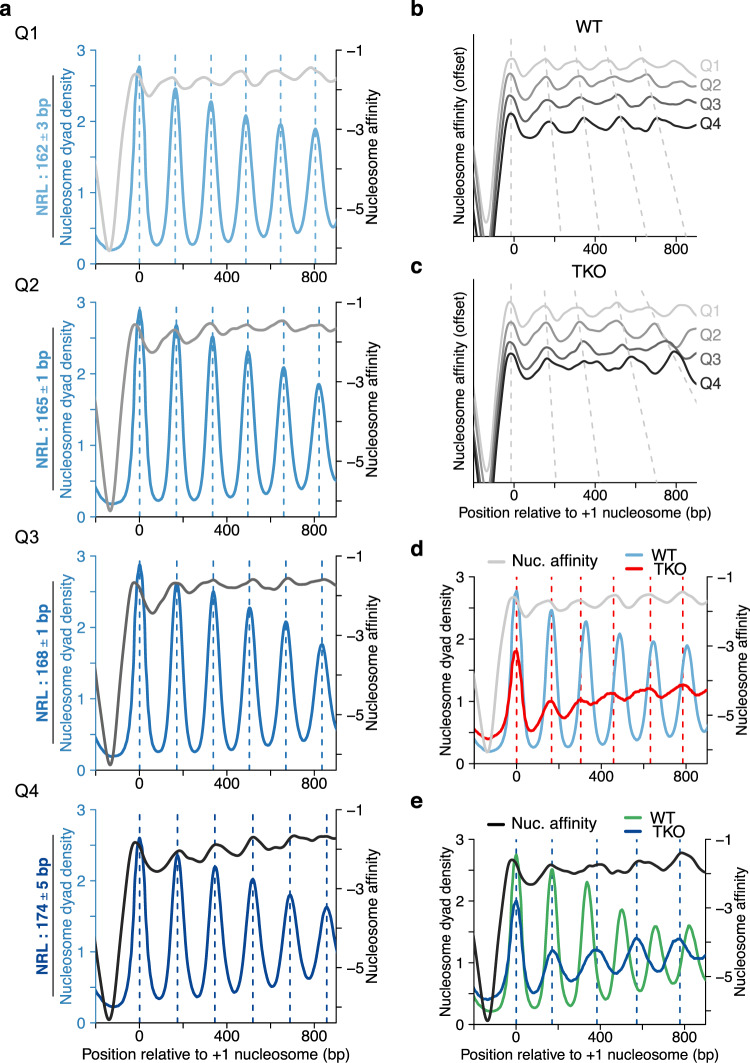
DNA sequence contributes to NRL determination. **a** Comparison of gene-averaged MNase-seq data and calculated nucleosome affinities for WT cells. Genes were filtered (see “Methods” section), sorted by NRL and grouped into quartiles from Q1 (shortest) to Q4 (longest NRL). NRL values are mean NRLs and their SD. Nucleosome affinities were calculated using nuCpos^48^. **b**, **c** Nucleosome affinities for Q1–Q4 of WT (**b**; replotted from **a** or TKO cells (**c**). **d** Arrays in Q1 of WT cells assume a shorter NRL in TKO cells. This shorter NRL is more similar to the NRL predicted from nucleosome affinities. **e** The NRL of arrays in Q4 of TKO cells is broadly consistent with the NRL predicted from nucleosome affinities. The same genes assume a substantially shorter NRL in WT cells, with peaks in the WT MNase-seq data straying further away from the thermodynamically most stable positions.

Remodelers are believed to override DNA sequence-encoded nucleosome positions^25^. If true, DNA sequence should not influence NRL. To test this prediction, we sorted genes by the NRL observed in WT cells and divided them into quartiles. For each quartile, we calculated the average nucleosome affinity (Fig. 6a)^48^. Evenly spaced peaks emerged in the average affinity profiles in all quartiles, and the peak-to-peak distances increased per quartile (Fig. 6a, b; dashed lines). To rule out that this was the case simply because the affinity algorithm was trained on WT nucleosome data, we performed the analogous analysis on TKO and TKO-Pol II datasets (Fig. 6c and Supplementary Fig. 6a, b). Moreover, we validated results with an alternative nucleosome prediction algorithm^46^ (Supplementary Fig. 6c, d).

Notably, peak positions between MNase-seq and the nucleosome affinity profiles largely superimposed in quartiles 2–4 in WT and in all quartiles in TKO and TKO-Pol II (Fig. 6a and Supplementary Fig. 6a, b). These results suggest that the DNA sequence influences NRL, and it does so even in presence of remodelers for the majority of genes, contrary to the expectation.

Nevertheless, MNase- and affinity profiles did not match well for genes with the smallest NRL (Q1) in WT (Fig. 6a). Here, the thermodynamically preferred NRL was even shorter than the measured one. The overlap improved when we plotted the MNase profile for the same genes from TKO cells (Fig. 6d). This observation suggests a direct contribution of one or more remodelers missing in TKO cells in overriding short, DNA-encoded NRLs.

TKO cells also possess genes with unusually long NRLs^10^. A superimposition of MNase and nucleosome affinity profiles of these genes (Q4) shows overlapping peaks, suggesting that the large NRL in TKO cells is at least in part DNA-sequence encoded. MNase profiles from WT cells for the same genes, however, showed a smaller NRL (Fig. 6e). We therefore suggest that ISWI and/or Chd1 remodelers can override and drastically shorten long, DNA-sequence encoded NRLs. By decreasing long and increasing short sequence-encoded NRLs, these remodelers conceivably contribute to equalizing linker lengths across the genome.

### Cells with regular nucleosome arrays experience less genotoxic stress

With ISWI, Chd1, and INO80 remodelers, the cell has evolved three families of spacing remodelers capable of generating nucleosome arrays. The functional relevance of nucleosome spacing, however, remains unknown. We hypothesized that arrays maintain genomic integrity by protecting the DNA from external insults.

**Fig. 7:**
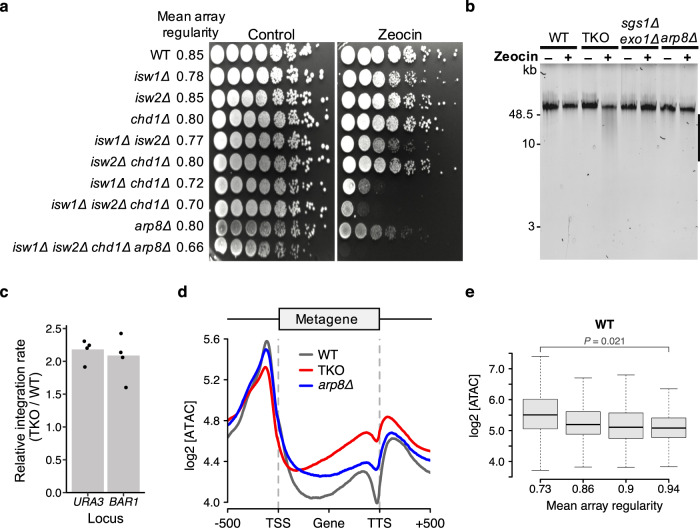
ISWI and Chd1 remodelers protect the genome from genotoxic stress. **a** Growth assay for indicated yeast strains on YPAD with and without Zeocin (100 µg/ml). **b** Zeocin-induced fragmentation of genomic DNA (bar) for the indicated strains. Cells were treated with deionized water (−) or Zeocin (+; 1 mg/ml) for 10 min. See Supplementary Fig. 7b for a biological replicate. **c** The TKO strain endures higher levels of ectopic recombination than the WT. Homologous recombination tested at two genomic loci (*URA3*, *BAR1*). Dots represent individual replicates from four independent experiments. Source data are provided as a Source Data file. **d** Metagene plot of ATAC-seq signals for WT, TKO, and *arp8*Δ cells from four independent replicates. **e** The number of ATAC-seq insertions into gene bodies anti-correlates with array regularity in WT cells. Genes were filtered for an absolute nucleosome occupancy between 0.78 and 0.88^66^, sorted by array regularity measured in WT cells and then divided into four quartiles. *P*-value (*P*) represent statistical analyses performed with two-tailed paired Welch’s *t*-test on the mean values of two replicates. Central line in box plot indicates the median, the box shows the interquartile range, and whiskers indicate data points within 1.5 times of the interquartile range.

First, we checked whether array regularity anticorrelates with susceptibility to DNA damage. We tested the stress response in cells that lack combinations of remodelers against zeocin, methyl methanesulfonate (MMS), hydroxyurea (HU), or ultraviolet (UV) radiation. Zeocin is a radiomimetic agent that generates free radicals and induces double-strand (ds) breaks. MMS and HU induce genome instability by inhibiting replication fork progression and depleting dNTPs, respectively^49^, whereas UV induces the formation of pyrimidine dimers. We found that combined loss of ISWI and Chd1 remodelers made cells highly susceptible to zeocin, MMS, and HU but not to UV (Fig. 7a and Supplementary Fig 7a). The growth defects of the mutant strains largely anticorrelated with the average array regularity of those cells, consistent with the notion that regular nucleosome arrays protect against DNA damage. Cells lacking *ARP8* were an outlier under HU and MMS stress as Arp8 has additional roles during replication and DNA repair^38,50^.

Strikingly, a brief exposure with zeocin (10 min) led to strong fragmentation of genomic DNA in TKO cells but much less so in WT cells and cells with defects in DNA repair (*sgs1*Δ *exo1*Δ^51^ and *arp8*Δ^52^) (Fig. 7b and Supplementary Fig. 7b). Defective DNA repair is therefore unlikely to account for the DNA-fragmentation phenotype of TKO cells; instead TKO cells may be naturally more prone to elevated DNA damage. Of note, genes covered with more regular nucleosome arrays are also less likely to suffer from naturally occurring DNA ds breaks during meiosis in WT cells (Supplementary Fig. 7c, d). These observations are consistent with even spacing of nucleosomes protecting against DNA damage.

Second, we tested whether cells with unevenly spaced nucleosomes might endure higher levels of ectopic DNA recombination. Homologous recombination rates^53^ at two genomic loci were ~two-fold larger in TKO compared to WT cells (Fig. 7c and Supplementary Data 2). The TKO mutation also elevated recombination rates when introduced into the *arp8Δ* background (Supplementary Fig. 7e), a genetic background that is known to recombine DNA more slowly than WT^53^. In summary, the presence of ISWI and Chd1 factors tend to suppress recombination. An attractive model is that they do so by keeping the genome evenly coated with nucleosomes, thus limiting DNA accessibility. Not all spacing factors suppress recombination, however. INO80, for instance, promotes recombination^52,53^, presumably through specialized mechanisms that are independent of spacing.

Third, we followed up on the hypothesis that spacing remodelers limit DNA accessibility. If true, transposon integration rates would be elevated in cells with higher levels of irregularly spaced nucleosome arrays. To measure transposon integration rates, we performed ATAC-seq in WT, TKO, and *arp8*Δ cells. Consistent with the model, gene bodies in TKO cells received higher levels of ATAC integrations than WT cells (Fig. 7d). Cells lacking *ARP8* showed an intermediate phenotype, consistent with an intermediate regularity of arrays in these cells (Fig. 7d).

In support of unevenly spaced arrays attracting transpositions, transposition rates over gene bodies tended to be higher for gene quartiles that have lower array regularity in WT cells, both in vivo (Supplementary Fig. 8a)^54^ and in ATAC-seq experiments (Fig. 7e). This trend was independent of transcription (Supplementary Fig. 8b), arguing against models in which ATAC rates increase simply because transcription disrupts the structural integrity of nucleosomes^55^ or temporarily increases access to DNA.

We confirmed that genes covered by unevenly spaced arrays also attracted higher ATAC integrations in *arp8Δ* cells (Supplementary Fig. 8c). This trend was not observable anymore in TKO cells probably because measurement of array regularity becomes noisy in cells with globally disrupted arrays. Nevertheless, given that most genes in WT have high regularity, the drop in array regularity caused by the TKO mutation can be estimated. We therefore asked if genes with a larger drop in regularity caused by the TKO mutation sustained more ATAC integrations, which tended to be the case. Similar results were obtained for *ARP8* deletion (Supplementary Fig. 8d, e). In conclusion, a model emerges in which regular arrays protect against transpositions.

Lastly, we asked whether irregular nucleosome arrays could contribute to cryptic transcription. In support of this possibility, cells with disrupted array structure, such as TKO cells and cells with defects in Spt6 and FACT, are known to experience elevated levels of cryptic transcription^23,56^. Moreover, genes that are covered with less regular nucleosome arrays also harbor more cryptic TSSs in WT cells (Supplementary Fig. 8f). The data are consistent with regular nucleosome arrays dampening cryptic transcription arising from the gene body.

## Discussion

The nucleosome landscape is shaped by numerous nuclear processes including a variety of nucleosome remodeling complexes and the transcription machinery. It is an experimental challenge to cleanly disentangle the effects of any one of these actors on the nucleosome architecture due to their strong functional redundancy. Here, we radically diminished redundancy of factors implicated in the biogenesis of the nucleosome landscape by simultaneously deleting or depleting Chd1, ISW1, ISW2, INO80, and components of the transcription machinery. This strategy helped us to isolate the role of transcription and led to the discovery that the INO80 complex can shape the canonical nucleosome architecture genome-wide.

### The mechanism of INO80-mediated spacing

We capitalized on our ability to isolate INO80 activity in the physiological environment of a cell to study its function and dissect its mechanism, an endeavor that traditionally is carried out in vitro. We find that the INO80 complex helps position the +1 nucleosome and directly or indirectly leads to nucleosome spacing (Figs. 3 and  5a). It can do so throughout the genome.

How does INO80 induce nucleosome spacing? In the simplest model, INO80 positions only the +1 nucleosome, which leads to better phasing of arrays and thereby allows their detection. In an alternative but mutually not exclusive model, INO80 can actively space arrays. The latter possibility is supported by our Arp8 results. Upon *ARP8* deletion, the NRL sharply decreases (Fig. 4d). This observation is consistent with a role of the Arp8 module in linker-length sensing but cannot readily be explained by INO80 only positioning the +1 nucleosome. We therefore suggest that INO80 can actively perform nucleosome spacing in vivo, consistent with observations in vitro^16,26,57^. However, it would be important to confirm the suggested spacing activity of INO80 and in fact all other spacing remodelers with technology that directly visualizes the distances between nucleosomes on individual DNA molecules^58,59^.

Why does INO80 need Arp8 for spacing? Arp8, conserved from yeast to mammals, is thought to sense the presence of linker DNA^18^. Deprived of this ability, Arp8-less Ino80 may not be able to space nucleosomes any longer^18^ which could contribute to lower overall nucleosome regularity in *ARP8*-deficient cells (Fig. 4d, e). The yeast-specific Nhp10 module, on the other hand, is not a critical component for INO80-mediated spacing (Fig. 4b). We could imagine, however, that yeasts evolved Nhp10 to optimize the efficiency of INO80-mediated spacing or help INO80 to space arrays in special situations, for example during replication or DNA damage^60^.

### Transcription is disruptive to nucleosome arrays

Active transcription has been suggested to be critical for the biogenesis of phased regular nucleosome arrays over genes^25^. Our data instead support an overall disruptive effect (Fig. 2). The disruptive effect may also be conserved in higher organisms because array regularity tends to be lower in highly transcribed *Drosophila* genes^61^. We propose that spacing remodelers counteract the disruptive effect of transcription. The opposing activities from nucleosome-organizing and nucleosome-disrupting factors could provide opportunities for chromatin-based regulation.

How transcription destroys array regularity remains unclear. One model posits that elongating Pol II evicts histones^62,63^. Several considerations argue against this model. First, we observe disruption of nucleosome regularity by the transcription machinery throughout the genome, not only at heavily transcribed genes (Fig. 2b, d). Second, loss of histones appears to precede transcription, not be caused by it^64,65^. Third, nucleosome occupancy over genes does not correlate with their transcriptional activity^66^. A model that would be consistent with available data invokes the elongation machinery to reposition nucleosomes. Indeed, we and others^34,67^ observed an increase in NRLs upon Pol II depletion (Supplementary Fig. 3a), suggesting that the transcription machinery moves nucleosomes upstream of their original locations. Such an activity, if not counteracted by remodelers, would over time destroy array regularity.

### Biogenesis of the nucleosome landscape

Besides transcription, DNA replication and damage are disruptive to the nucleosome landscape^5^. Our findings extend previous models of how cells reestablish the nucleosome landscape^24,26^.

We envision four contributing processes that jointly sculpt the nucleosome landscape^68^. First, nucleosomes preferentially accumulate on DNA sequences that inherently possess a high affinity to nucleosomes. We observe such an accumulation in three quarters of the WT genome and in remodeler-depleted and histone-depleted cells. Despite drastically different nucleosome organization in these mutant cells, we did not observe nucleosomes being substantially enriched or depleted from sites with high nucleosome affinity (Supplementary Fig. 6a, b). We thus suggest that the nucleosome affinity landscape flexibly allows for several thermodynamically equivalent nucleosome configurations.

Another, second process helps form the NFR. Nucleosome destabilizing DNA sequences enriched at the NFR, GRFs and the RSC remodeler play an important roles in keeping nucleosomes away from NFRs^8^.

A third process positions the +1 nucleosome. RSC pushes the +1 nucleosome away from its highest affinity site further into the gene body (Table 1). Without the action of INO80 and ISW2, the +1 nucleosome would continue to slide deeper into the gene body. These results suggest that RSC engages in a tug-of-war with INO80 and ISW2, which could lead to sharpening of the +1 position^3^. H2A.Z on the other hand has no observable effect on +1 positioning, in line with prior results^69^.

**Fig. 8:**
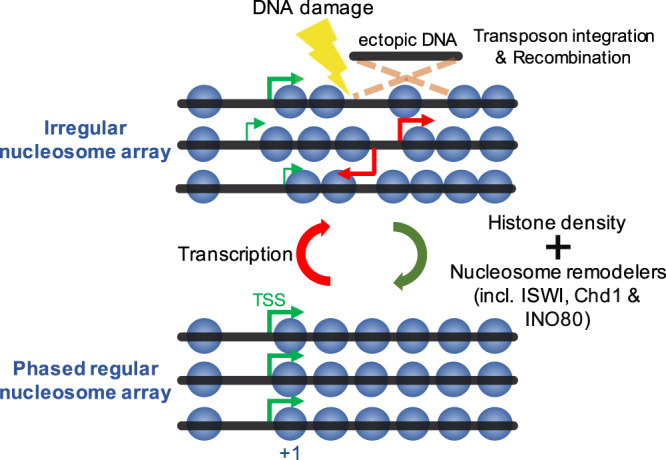
Model for biogenesis and function of regular nucleosome arrays. Transcription is strongly disruptive to nucleosome arrays and it takes ISWI-, Chd1- and/or INO80- remodelers to reinstate array architecture. Proper histone density is indispensable for spacing as neither DNA sequence-based nucleosome positioning nor the clamping activity of remodelers suffices. Remodelers also position the +1 nucleosome, thereby sharpening the site of transcription initiation (green arrows). Regularly spaced nucleosome arrays prevent cryptic transcription (red arrows), DNA damage, ectopic recombination and transposon integration within the gene body.

In the fourth process, remodelers of the ISWI, Chd1 and INO80 families space nucleosomes in gene bodies (Fig. 8). They can override DNA-sequence encoded nucleosome spacing, particularly over genes that have extreme sequence-encoded NRLs. The NRL distribution thereby considerably sharpens. In three quarters of all genes, however, nucleosomes end up again on positions with equally strong nucleosome affinities. DNA sequence therefore codetermines NRLs in most of the genome, and even ATP-dependent remodelers cannot completely level the thermodynamic landscape for nucleosomes.

Our nucleosome depletion experiments suggest that at least one ISWI or Chd1 remodeler possesses a clamping activity, holding nucleosomes at a close distance even when nucleosome concentrations are diminished. Note that this result does not rule out the linker length equilibration mechanism^14^. In fact, the clamping activity could be a manifestation of linker length equilibration provided that the remodeler can measure linker lengths only over short ranges. Nevertheless, the observed clamping activity is too weak to overcome the entropic forces acting on nucleosomes upon their depletion. The biogenesis of arrays therefore also requires WT-like nucleosome densities (Figs. 1a and 8).

The physiological NRL of ~165 bp has been suggested to result from a competition between ISWI and Chd1 remodelers, with Chd1 promoting narrower and ISW1 wider spacing^10^. Future models should also take INO80 into account and consider the possibility that remodeler activities may not be fully independent of each other.

### The function of nucleosome arrays

Why do eukaryotic cells evenly space nucleosomes? We speculate that even spacing dampens cryptic transcription, perhaps by occluding cryptic TSSs in gene bodies, and thereby forcing the transcription machinery to initiate from canonical TSSs (Fig. 8 and Supplementary Fig. 8f).

In addition, we propose that regularly spaced nucleosome arrays may counteract the integration of transposons (Figs. 7d and 8). An even spacing of nucleosomes could for example prevent occasional exposure of a large enough stretch of naked DNA that mobile elements or retroviruses would exploit to integrate into the genome. Similar considerations would explain our observation that TKO cells with their irregular nucleosome array structure suffer from higher homologous recombination rates than WT cells (Fig. 7c).

Furthermore, we hypothesize that spacing remodelers may have evolved to suppress genotoxicity from DNA damaging events (Fig. 8). This model would explain why the DNA of cells lacking ISWI and Chd1 remodelers very quickly fragments in response to DNA-damaging agents (Fig. 7b), and why genes with irregularly spaced nucleosomes are particularly prone to DNA double strand breaks (Supplementary Fig. 7c, d).

Protection against mobile elements, retroviruses, homologous recombination, cryptic transcription, and DNA damage may have been powerful evolutionary advantages that ensured retention of spacing remodelers throughout the eukaryotic domain.

## Methods

### Yeast strain generation

All yeast strains used in this study are derived from the W303 background (Supplementary Data 1). To validate gene deletions and tagging, all loci of interest were tested by PCR (for oligonucleotides, see Supplementary Data 2).

To delete *ISW1*, *ISW2*, and *CHD1* from anchor-away cells, HHY170^30^ was mated to YTT227^27^ and haploids were obtained via tetrad dissection. Other deletions in all anchor-away strains were done via direct transformation. For TBP and INO80 depletion experiments, *SPT15* and *INO80* genes were C-terminally tagged with a FRB and GFP fusion construct. To obtain a TKO histone depletion strain, DY5734^70^ was mated to YTT227 and haploids were obtained via tetrad dissection. The Ycp50 *HHT2-HHF2* plasmid was switched to pRS413 Gal1-10 *HHT2*-*HHF2* (pFMP519) via 5-FOA selection.

### Yeast growth conditions

To deplete RNA Pol II, INO80, or TBP, cells were grown to OD 0.2–0.3 before addition of vehicle (90% ethanol, 10% Tween-20) or rapamycin (1 μg/ml final concentration, dissolved in vehicle).

For histone depletion experiments, cells were initially grown overnight to OD 1.0, washed with pre-warmed SC media without carbon source and dissolved in pre-warmed SC +2% glucose media to OD 0.5. Cells were grown at 30 °C for 3 h and harvested for MNase-seq. Cells grown in parallel with 2% galactose instead of glucose were used as controls for histone depletion.

### Yeast nuclei preparation and MNase digestion

Yeast nuclei were prepared largely as described^71^. Strains were grown overnight to OD 0.8–1.0 in YPAD media. Cells were harvested (3000×*g*, 8 min, 4 °C) and washed once with cold water. The pellet was weighed (wet weight), resuspended in two volumes of preincubation solution (0.7 M ß-mercaptoethanol, 28 mM EDTA pH 8.0) and shaken at 30 °C for 25–30 min. Cells were washed with 40 ml ice-cold 1 M sorbitol and resuspended in five volume of 1 M sorbitol, 5 mM ß-mercaptoethanol. One milligram of freshly dissolved Zymolyase was added/g wet weight and incubated at 30 °C for 20–30 min until the OD 600 reading was decreased to 80–90% of the initial OD. Cells were also checked under the microscope for appearance of 80-90% ghosts. Spheroplasts were collected by centrifugation (2500×*g*, 5 min, 4 °C) and washed with 40 ml ice-cold 1 M sorbitol. Spheroplast pellets were resuspended in 7 ml/g wet weight Ficoll buffer (18% Ficoll, 20 mM KH_2_PO_4_ pH 6.8, 1 mM MgCl_2_, 0.25 mM EGTA, 0.25 mM EDTA). Nuclei were centrifuged (12,000×*g*, 30 min, 4 °C) and stored at −80 °C.

For MNase digestion, nuclei were thawed on ice for 10 min, washed once with 8 ml MNase digestion buffer (15 mM Tris-Cl pH 7.5, 50 mM NaCl, 1.4 mM CaCl_2_, 0.2 mM EGTA pH 8.0, 0.2 mM EDTA pH 8.0, 5 mM ß-mercaptoethanol), dissolved in 1 ml MNase digestion buffer and divided into five aliquots. An increasing amount of MNase (1, 2, 4, 8, and 16 μl from 0.58 U/μl stock (Sigma, Cat# N5386); dissolved in 10 mM HEPES-KOH pH 7.6, 50 mM NaCl, 1.5 mM MgCl_2_, 0.5 mM EDTA, 10% Glycerol, 1 mM DTT, 0.2 mM PMSF) was added, mixed, and incubated for 20 min, 37 °C. MNase digestion was stopped by adding 35 μl of quenching solution (10 mM EDTA, 1% SDS, 50 mM Tris-Cl pH 8.5). Three hundred microgram Proteinase K (from 10 mg/ml stock) was added and incubated at 37 °C for 30 min. Afterwards, 70 μl of 5 M NaClO_4_ was added. The samples were extracted with Phenol:Chloroform:Isoamyl alcohol (25:24:1), then with Chloroform:Isoamyl alcohol (24:1) and precipitated with 100% ethanol. Pellets were washed with 70% ethanol and dissolved in 250 µl TE buffer. RNA was removed by adding 1 µl RNase A (from 10 mg/ml stock) and incubation at 37 °C for 60 min. DNA was precipitated by adding 0.2 M NaCl (final concentration) and 0.7 volumes of Isopropanol, washed with 70% ethanol and dissolved in 50 μl TE buffer. To visualize the digestion degree, 25 μl of DNA was mixed with 3 μl of loading dye (0.5% Orange G, 50% glycerol) and electrophoresed on a 1.7% low-melt agarose gel. Whenever possible, samples showing 70% mono-nucleosome, 20% di-nucleosome, 10% tri-nucleosome bands were used for high-throughput sequencing.

### Cell cycle profile

To measure cell cycle progression, 10^7^ cells were harvested, resuspended in 1 ml of 50 mM Tris pH 8.0, 70% ethanol and incubated overnight at 4 °C. Cells were washed with 50 mM Tris pH 8.0 and incubated with 520 μl of RNase solution (500 μl of 50 mM Tris pH 8.0, 20 μl of 10 mg/ml RNase A) overnight at 37 °C. Cells were treated with 220 μl Proteinase K solution (200 μl 50 mM Tris pH 8.0, 20 μl 10 mg/ml Proteinase K) for 30 min at 50 °C. Cells were and resuspended in 500 μl of 50 mM Tris pH 8.0 and sonicated for 15 s (Bioruptor Pico). Fifteen microliter cells were stained with 285 μl of SYTOX solution (10 mM Tris pH 8.0, 1:10,000 dilution, Cat# S7020 Life Technologies) and analyzed with BD FACSCanto or BD Fortessa (Core Facility Flow Cytometry Biomedical Center, LMU Munich).

### G1 cell cycle arrest

Cells were grown to OD 0.2 in YPAD media and alpha factor (10 μg/ml final concentration; Hölzel Diagnostika Cat# RP01002)) added. After 1 h incubation, the same amount of alpha factor was added, and cells were incubated for one more hour. Cells were again supplemented with alpha factor (5 μg/ml final concentration), divided equally into two flasks and treated with either vehicle or rapamycin.

### RNA Pol II ChIP

Cells were grown to OD 0.4 in 500 ml YPAD media and treated with vehicle or rapamycin. Cells were crosslinked (1% formaldehyde, 10 min, 25 °C) and quenched with 125 mM glycine. Cells were harvested, washed with ST buffer (20 mM Tris-HCl pH 7.5, 100 mM NaCl) + protease inhibitors (PI; 1 μg/ml Aprotinin, 1 μg/ml Leupeptin, 1 μg/ml Pepstatin A, 1 mM PMSF). Cell walls were disrupted in FA-lysis buffer (50 mM HEPES pH 7.5, 140 mM NaCl, 1 mM EDTA pH 8.0, 1% Triton X-100, 0.1% Na Deoxycholate, PI) by bead beating (Precellys 24 homogenenizer). Chromatin was fragmented to an average size of 500 bp using Diagenode Bioruptor Pico for 20–25 cycles of 30″ on/30″ off in FA-lysis buffer + 0.25% SDS. SDS was diluted in the supernatant to 0.1% with FA-lysis buffer and incubated with 2 μg anti-Rpb3 antibody (Abcam Cat#1Y26; 2 μg/IP) for 2 h at 4 °C. Hundred microliter of pre-washed Pan-Mouse IgG Dynabeads were added and incubated overnight at 4 °C. Beads were washed twice with FA-lysis buffer, twice with FA-lysis + 360 mM NaCl buffer, twice with FA-wash 2 buffer (0.25 M LiCl, 0.5% NP40, 0.5% Na-deoxycholate, 1 mM EDTA, 10 mM Tris-Cl pH 8.0) and once with TE buffer. DNA was eluted in 50 μl TE buffer + 0.5% SDS at 65 °C for 1 h. RNA was removed with 1 μg RNase A (1 h at 37 °C) and DNA was de-crosslinked overnight by incubating with 10 μg Proteinase K at 65 °C. Immunoprecipitated DNA were quantified by qPCR using Fast SYBR master mix (Life Technologies, Cat# 4385618). For oligonucleotides see Supplementary Data 2.

### NGS library preparation and sequencing

We prepared sequencing libraries directly from the whole MNase digested samples, not from mononucleosomal DNA, to avoid artifacts arising from imprecise gel extraction. DNA fragments longer than 500 bp were removed using AMPure size selection as follows: 300 ng DNA was diluted in 50 μl 0.1× TE buffer. 0.65× volumes of AMPure XP beads were added. 1.92× volumes of Ampure XP beads were added to the supernatant in a new eppendorf. Beads were washed twice with 500 μl freshly prepared 80% ethanol, and DNA was eluted in 30 μl 0.1× TE buffer. Fifty nanogram of DNA was used for library preparation using NEBNext Ultra II DNA Library Prep kit for Illumina. Three to four PCR cycles were performed. Fragment lengths of ~270–280 bp were consistently observed. Libraries were sequenced on Illumina HiSeq for 50 cycles in paired-end mode.

### Western blot

Cells were grown overnight, reinoculated in fresh 10 ml YPAD to OD 0.1 and grown to OD 0.8. Extracts were prepared via the NaOH/TCA precipitation method. Blots were incubated overnight with primary antibodies in 5% skimmed milk + PBS with 0.1% Tween-20. Anti-FLAG M2 (Sigma F1804, 1:20,000), anti-Histone 3 (Abcam ab1791, 1:20,000) and anti-Histone 4 (abcam ab10158, 1:2000) primary antibodies were used. LI-COR IR secondary antibodies (IRDye 680RD Goat anti-Mouse, IRDye 800CW Goat Anti-Mouse, IRDye 800CW Goat Anti-Rabbit) 1:10,000 dilution and Odyssey IR imaging system were used for visualization.

### Spotting assay

Cells were grown overnight to near saturation and OD 600 was measured in technical replicates after 1:10 dilution in water. Cells were diluted to OD 1.0 in 200 µl water and 5-fold dilutions were generated. Seven microliter of these dilutions were spotted on YPAD supplemented with desired compound as necessary. Plates were incubated at 30 °C for 3 days. Assays were performed twice using two independent colonies of the mutant strains.

### DNA damage assay

Cells were grown overnight in 50 ml YPAD to OD 0.4–0.8. Log-phase cultures were diluted to OD 0.2 in 5 ml YPAD media supplemented with zeocin (1 mg/ml final concentration). Cells were incubated at 30 °C for 10 min with gentle shaking, harvested, washed with 5 ml ice-cold water and dissolved in 200 µl DNA extraction buffer (0.9 M Sorbitol, 50 mM Na-Pi pH 7.5, 140 mM ß-Mercaptoethanol, 15 mM sodium azide). Cell walls were digested with Zymolyase (0.5 mg/ml, 15 min), followed by Proteinase K digestion (2 mg/ml, 15 min), both at 30 °C. Phenol: Chloroform: Isoamyl alcohol (25:24:1) extraction was performed once, followed by ethanol precipitation. Pellets were dissolved in 100 µl TE buffer supplemented with 5 µg RNase A and incubated for 30 min at 30 °C. Twenty microgram of DNA was separated by electrophoreses on a 0.6% (w/v) low-melt agarose gel (1× TBE, 2 V/cm, 4 h). The gel was stained with ethidium bromide (0.5 µg/ml) in 1× TBE for 15 min. Samples were pipetted only three times with ends of pipette tips cut off to minimize fragmentation arising from shearing during pipetting.

### Ectopic recombination assay

The ectopic recombination rate was determined as described^53^. An equal number of log phase cells (10^7^) were transformed. For insertions at the *ura3-1* locus, pRS406 was StuI-digested. For insertions at the *BAR1* locus, the HIS3 marker was amplified from pRS403 plasmid. As a negative control, undigested pRS406 or pRS403 plasmids (100 ng) were transformed. As a positive control, 100 ng of pRS416 or pRS413 plasmids were transformed in parallel. To calculate relative integration rates, the numbers of colonies obtained on StuI digested pRS406 or *HIS3* PCR product were first divided by the number of colonies obtained for their positive controls (pRS416 or pRS413, respectively). Normalized count for each replicate was divided by the normalized count observed in WT cell to obtain the relative integration rate.

### Cloning via Gibson assembly

*ARP8* including its native promoter and terminator was PCR-amplified from genomic DNA and cloned into the pRS416, yielding pFMP549. By inverse PCR, amino acids 2-197 were deleted (pFMP549). To clone the Gal1-10 *HHT2*-*HHF2* construct from the pRM102 plasmid^28^ into pRS413, the Gal1-10 *HHT2*-*HHF2* construct was PCR amplified and cloned via Gibson assembly, yielding pFMP519. For list of cloned plasmids, see Supplementary Table 1.

### ATAC-seq

The ATAC-seq protocol for yeast^72^ was provided by William Greenleaf and used with minor changes. Transposition reactions were performed in 25 µl tagmentation mix (1.25 µl Illumina Tagment DNA Enzyme (Cat# 20034197), 12.5 µl 2× Illumina Tagment DNA buffer, 11.25 µl water) for 15 min, 37 °C with gentle shaking. DNA was purified and PCR amplified in 50 µl reaction (10 µl tagmented DNA, 10 µl water, 2.5 µl each Nextera index i5 and i7 primers and 25 μl NEBNext High-Fidelity 2X PCR Master Mix) initially for five cycles. Then, qPCR was performed on the amplified samples to calculate the minimum number of cycles (usually 4) required to avoid over-amplification. Libraries were size selected aiming for final fragment size 100–600 bp.

### Data analysis

All MNase-seq experiments were performed in biological replicates using two independent colonies, except for Rpb1 anchor-away and Nhp10 deletion in WT backgrounds, for which published datasets exist^34,35,40^. Nuclei preparation and MNase digestion from two yeast colonies were performed on different days. Sequencing libraries were prepared in parallel for both colonies.

Biological replicates were first analyzed separately and found highly consistent. For final analysis, bam files of biological replicates were merged for further downstream analyses. All statistical analyses were performed in R.

Demultiplexing, mapping, and coverage—Fastq files from Illumina HiSeq were demultiplexed using Je demultiplex suite v1.0.6. Sequences were mapped to *S. cerevisiae* sacCer3 R64-1-1 genome using Bowtie2 v2.2.9 with default settings, except -X 500, –no-discordant, –no-mixed options. Bam files were created using samtools v1.3.1 with minimum mapping quality 2 and mitochondrial (chrM) and rDNA reads (chrXII 451000:469000) were removed. Nucleosome dyad coverage and bigWig files were generated by taking the center of 140–160 bp fragments and resizing to 50 bp at the dyad center. All samples were sub-sampled to 10 million reads to generate an equal number of reads. The sequencing coverage was normalized to reads per million.

Composite plot and heatmap—Genome coordinates and annotations for all genes, including the +1 nucleosome, were downloaded from ref. ^73^ A matrix aligned to the +1 nucleosome was generated using coverageWindowsCenteredStranded function in tsTools v0.1.2 (https://github.com/musikutiv/tsTools). Composite plots were created from the aligned matrix by calculating the mean signal for each base pair of all genes and normalized to the mean signal of the desired window. Heatmaps were also generated using the aligned matrix.

NRL and regularity score calculation—A MATLAB routine for calculating the NRL was generously provided by Răzvan Chereji and David Clark^10^. We adapted it in R (https://github.com/musikutiv/tsTools) with some modifications. For each gene, the genomic region at the +1 nucleosome was smoothed with a 75 bp smoothing window. The smoothed nucleosome patterns were cross correlated with a theoretical periodic pattern of Gaussian distributions with increasing repeat lengths. For each gene, the NRL was taken from the nucleosome pattern that resulted in the highest cross-correlation score. This cross-correlation score was used as an estimate for the array regularity, with higher coefficients indicating higher array regularity.

For 1 and 2 h Pol II depletion in TKO cells, 2 h depletion showed only minor increase in array regularity compared to 1 h depletion, suggesting that 1 h depletion leads to the majority of the increase in array regularity.

ATAC-seq analysis—Paired-end reads were processed and mapped to sacCer3 R64-1-1 genome using Bowtie2 v2.2.9 with default settings. A coverage vector was generated from the bam files taking fragments between 50 and 500 bp with equal number of reads (3 million) for each sample. A matrix aligned to the TSS and TTS^73^ were generated from the coverage vector and gene bodies were re-scaled to 1000 bp. For ATAC to array regularity correlation, reads mapping between TSS +100 bp and TTS −100 bp, and genes with mRNA longer than 300 bp and nucleosome occupancy between 0.78 and 0.88^66^ were considered.

Published datasets—RNA Pol II anchor-away, RSC and INO80 depletion and *nhp10*Δ MNase-seq datasets in the WT background were taken from refs. ^3,34,35,40,47^, and reprocessed with our bioinformatics pipeline. Rpb3 ChIP-seq datasets are from ref. ^10^. Bam files with fragment lengths between 50 and 300 were used to calculate average RNA Pol II occupancy (IP/input) over each gene using the bamR package (https://github.com/rchereji/bamR).

DANPOS + 1 nucleosome calling and fuzziness analysis—Bam files with fragment lengths between 140 and 160 bp were used as input. Nucleosomes were called using dpos command with -jw 5, -q 200, -m 1 parameters in DANPOS v2.2.2^43^. The first nucleosome called after the NDR coordinate^73^ of each gene was used as the +1 nucleosome and summit value for each gene was taken.

Simulated statistical positioning after histone depletion—Simulations with 10000 DNA fragments between 2000 and 2500 bp in length were carried out in MATLAB R2013b (The Mathworks). The +1 nucleosome was positioned on one end of each DNA fragment using a gaussian function (sigma of 25 bp) that approximates MNase-seq derived values. All other nucleosomes were successively placed on a random DNA fragment at a random position, provided that this position was not occupied already. Nucleosomes were modeled as hard spheres with a footprint of 146 bp. Random placement of nucleosomes continued until nucleosomes covered ~51% of available DNA. After that, 20% of all nucleosomes (picked at random, but excluding the +1 nucleosome) were dissociated again. The dissociated nucleosomes were placed on the DNA fragments again as above, and the dissociation and placement cycle was repeated another eight times. Control simulations with lower (~43%) and higher (~60%) coverage revealed +2 peak positions within ~10 bp of each other.”

Nucleosome affinity prediction using nuCpos—Nucleosome affinities were calculated with the Histone Binding Affinity (HBA) function in nuCpos^48^, which provides a histone binding affinity score for a given 147 bp DNA sequence. The genome was divided into 147 bp sequences with a 1 bp sliding window step size. The HBA signal was aligned to +1 nucleosome^73^ and smoothed using smooth.spline function (spar = 0.4). Smoothing using the rollmean (Zoo package) or sgolayfilt (signal package) functions yielded similar results. Only genes with discernible array structures were selected by filtering for array regularities >0.5 and NRLs between 150 and 200 bp. Each quartile represents the average signal of 786, 502, and 688 genes in WT, TKO, and TKO-Pol II samples, respectively.

Nucleosome positioning sequence (NPS) from Ioshikhes et al.^46^. NPS data for coordinates −931 to +528 relative to start codons^73^ were re-aligned to the positions of the +1 nucleosomes. Genes with array regularity >0.5 and NRLs between 150 and 200 bp were used. NPS data were smoothed using rollmean (Zoo package v1.8-5) with step size 51.

Correlations of array regularity with cryptic TSSs, ds breaks and transposon integrations—To correlate cryptic TSS^32^, transposase insertions^54^ and Spo11-induced and topoisomerase 2-induced ds breaks^74^ to array regularity, counts mapping in the region TSS +100 to TTS −100 bp were summed for each gene and divided by the analyzed length. Genes were sorted by array regularity and divided into quartiles. For transposase insertion data^54^, only nonessential genes were included as insertions in essential genes were mostly lethal. Genes longer than 500 bp and nucleosome occupancy between 0.78 and 0.88^66^ were used for these analyses.

### Reporting summary

Further information on research design is available in the Nature Research Reporting Summary linked to this article.

## Supplementary information

Supplementary Information

Description of additional Supplementary File

Supplementary Dataset 1

Supplementary Dataset 2

Reporting Summary

## Data Availability

The data that support this study are available from the corresponding author upon reasonable request. The next generation sequencing data have been deposited at the GEO under accession number GSE141007. Publicly available datasets were retrieved using the following GEO accession number: GSE73337, GSE112465, GSE49512, GSE115412, and from Sequence Read Archive: SRP051897. Source data are provided with this paper.

## Source data

Source Data
