# Supplementary material for: The biogenesis and function of nucleosome arrays
Source: Nat Commun. 2021 Dec 1;12:7011. doi: 10.1038/s41467-021-27285-6 (PMC8636622; doi:10.1038/s41467-021-27285-6)
Supplement: Supplementary file 1 — Supplementary Information [file 41467_2021_27285_MOESM1_ESM.pdf]

Supplementary Information

The biogenesis and function of nucleosome arrays

Ashish Kumar Singh[1], Tamás Schauer[†,2], Lena Pfaller[†,1,3], Tobias Straub[2], Felix Mueller-Planitz[1,4*]

[1]Molecular Biology, Biomedical Center, Faculty of Medicine, Ludwig-Maximilians-Universität München, 82152 Planegg-Martinsried, München, Germany

[2]Bioinformatics Unit, Biomedical Center, Faculty of Medicine, Ludwig-Maximilians-Universität München, 82152 Planegg-Martinsried, München, Germany

[3]Present address: Novartis Institutes for BioMedical Research, CH-4056 Basel, Switzerland

[4]Institute of Physiological Chemistry, Faculty of Medicine Carl Gustav Carus, Technische Universität Dresden, Fetscherstraße 74, 01307 Dresden, Germany.

[†]These authors contributed equally to this work

[*]Correspondence: felix.mueller-planitz@tu-dresden.de

Table of Contents

Supplementary Figures 1

Supplementary Table 1 12

Supplementary References 13

SUPPLEMENTARY FIGURES

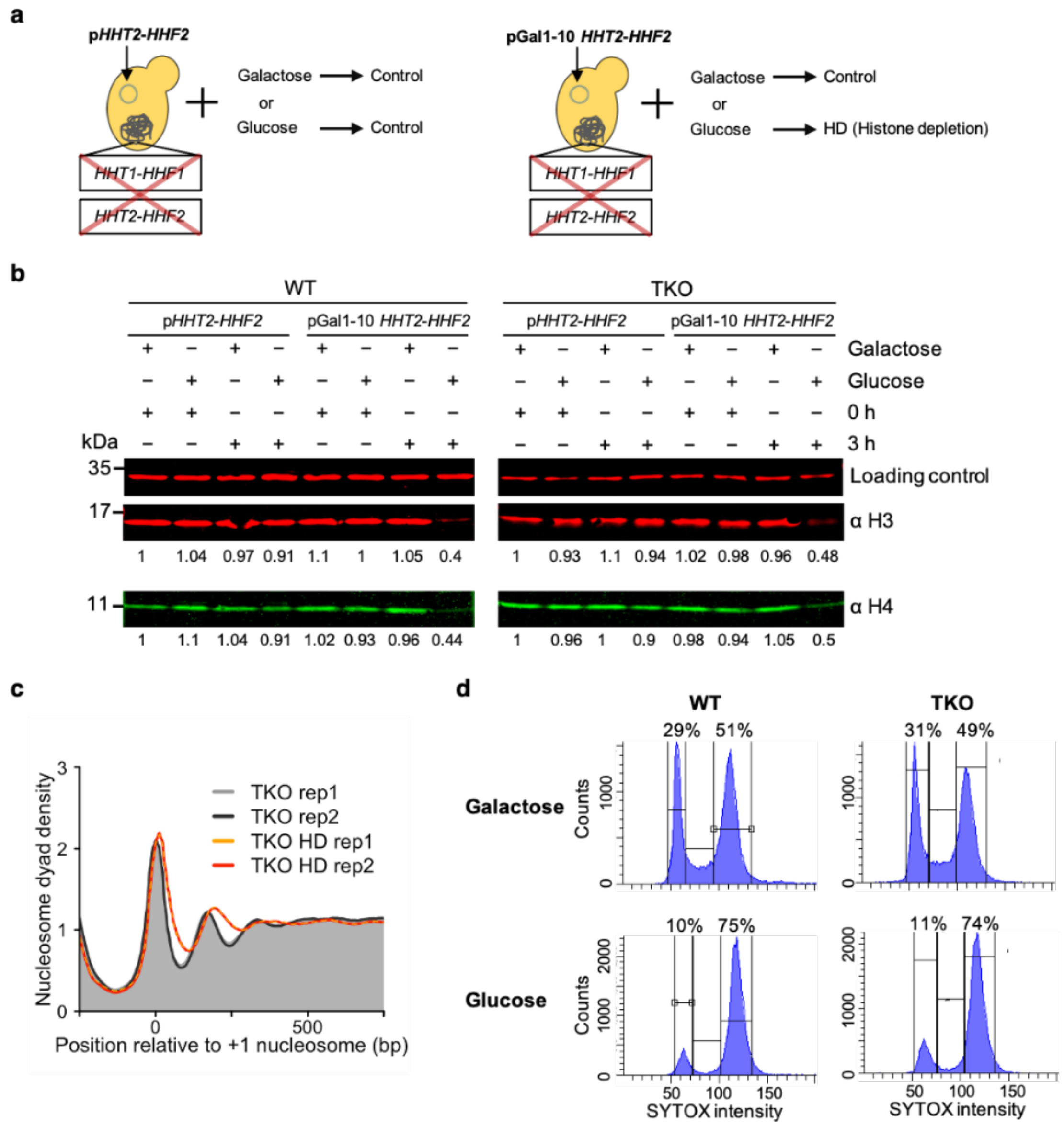

Supplementary Figure 1: Histone depletion in WT and TKO cells. a Scheme of the HD system[1]. Genomic copies of histone H3 and H4 genes are deleted and replaced with plasmid-borne copies that either remain under control of their native promoter (pHHT2-HHF2) or a galactose-inducible promoter (pGal1-10 HHT2-HHF2). Glucose-mediated repression of cells containing pGal1-10 HHT2-HHF2 leads to histone depletion. All other conditions serve as negative controls. **b** Glucose-mediated repression leads to reduction of H3 and H4 protein levels of ≥50% in WT and TKO cells. Numbers are relative expression levels between strains and conditions and represent the mean of two biological replicates. Values are normalized to a cross-reacting band detected by the anti-FLAG M2 antibody, which served as a loading control. Values were further normalized to H3 and H4 amounts measured in the left-most lane of each blot (pHHT2-HHF2 grown in galactose). Source data are provided as a Source Data file. **c** Composite plots showing nucleosome organization in TKO and TKO HD cells in two biological replicates. **d** Cell cycle analysis by flow cytometry before (galactose) and after histone depletion (glucose) in WT and TKO cells. Cells were grown in galactose- or glucose-containing minimal media for 3 h as in **b**. Values represent percentage of cells in the G1 and G2/M phases.

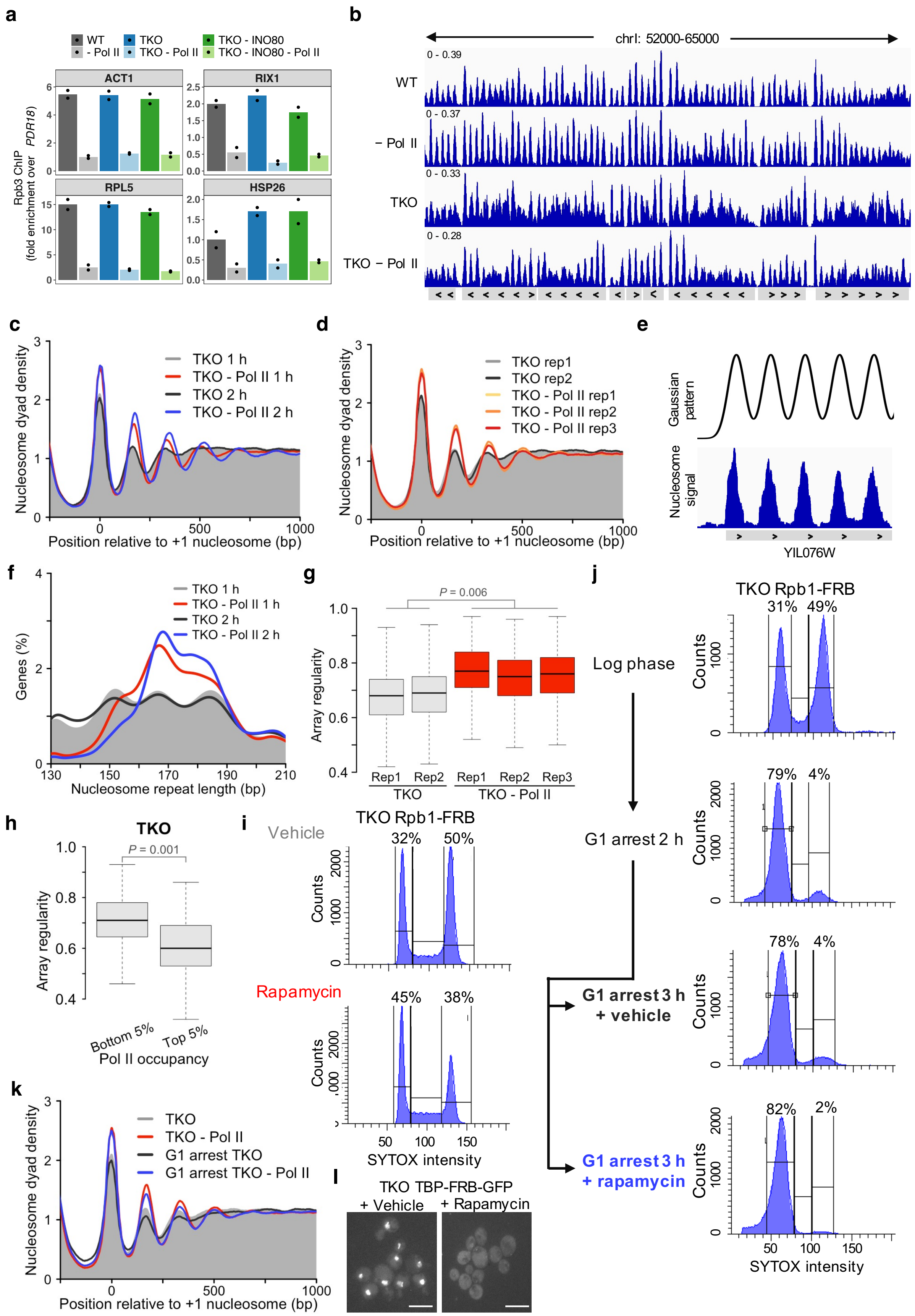

Supplementary Figure 2: RNA Pol II depletion increases array regularity in TKO cells. a Pol II is efficiently depleted from four representative genes upon addition of rapamycin in Rbp1-FRB tagged cells. ChIP-qPCR analysis of Rbp3 immunoprecipitation in WT, TKO and INO80 depleted TKO cells

before and after Pol II depletion. Signals were normalized to immunoprecipitated DNA of the lowly expressed *PDR18* gene for each strain. Errors: Minimum and maximum values from two independent measurements. Source data are provided as a Source Data file. **b** Genome browser view of MNase-seq in the indicated yeast strains. **c** Nucleosome organization upon Pol II depletion for 1 h and 2 h in TKO cells. TKO controls also harbored the FRB-tagged Rpb1 but were treated with vehicle. 1 h depletion data are replotted from Figure 2a. **d** Biological replicates for Figure 2a. **e** To obtain NRL and regularity over each gene, MNase-seq profiles over each gene are cross-correlated to Gaussian patterns of varying NRLs. The best-fitting Gaussian pattern provides an estimate of the NRL, and the correlation coefficient an estimate of the regularity. **f** NRL distribution of strains in **c**. Peak maxima after Pol II depletion are at 167 bp (1 h) and 168 bp (2 h), respectively. **g** The median array regularity increases in biological replicates (Rep) upon Pol II depletion in TKO cells. P-value (*P*) represent statistical analyses performed with two-tailed Welch's t-test on the mean values of individual replicates. **h** Array regularity of bottom and top 5% Pol II-bound genes in TKO cells. P-value (*P*) represent statistical analyses performed with two-tailed Welch's t-test on the mean values of two replicates. Central lines in box plots in (g, h) indicate the median, the box shows the interquartile range, and whiskers indicate data points within 1.5 times of the interquartile range. **i** Cell cycle analysis upon Pol II depletion in Rbp1-FRB tagged TKO cells. Cells were treated with vehicle or rapamycin for 1 h. **j** Cell cycle analysis of Rbp1-FRB tagged TKO cells before (log phase) and after treatment with alpha-factor for 2 h (G1 arrest 2h). Cells were then treated with vehicle or rapamycin for 1 h. Values represent percentages of cells in G1 and G2/M phases. **k** Nucleosome organization in cycling and G1-arrested Rbp1-FRB tagged TKO cells. Pol II was depleted for 1 h after a 2 h G1 arrest. **l** Live-cell imaging of FRB-GFP-tagged TBP shows its 1 h depletion from the nucleus. Images are representative of two biological replicates. Scale bar 5 µm.

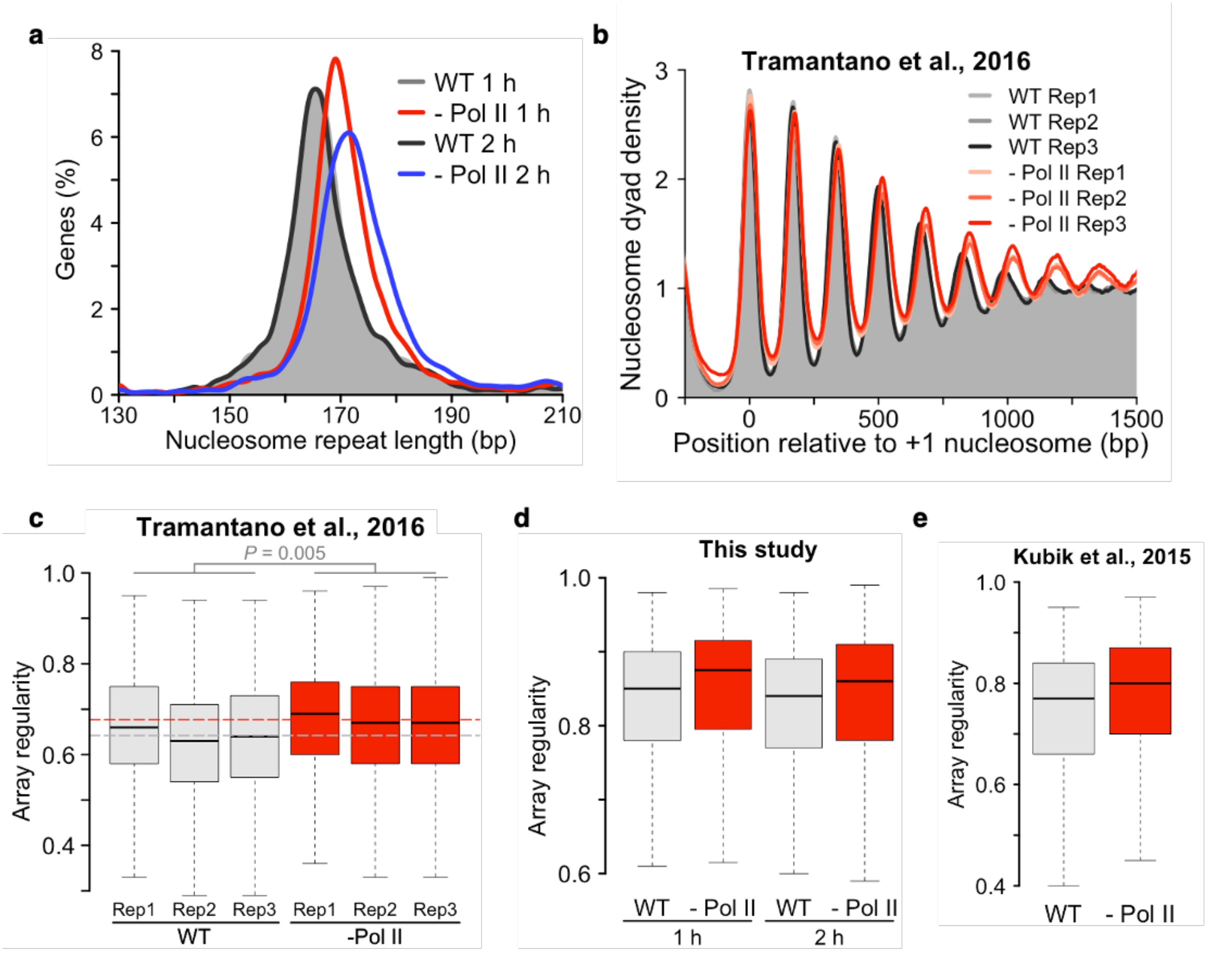

Supplementary Figure 3: RNA Pol II depletion in WT cells. a NRL distribution before and after Pol II depletion from WT cells. Peak maxima are at 169 bp (1 h depletion) and 172 bp (2 h), respectively. Control samples are Rpb1-FRB tagged; they were vehicle-treated for 1 h or 2 h and show peak maxima at 166 bp and 165 bp, respectively. **b** Nucleosome organization in Pol II depleted cells. Data from ref. 2. **c** Median array regularity (horizontal lines; average of three replicates) increases upon Pol II depletion in WT cell. Data from ref. 2. *P* value represent statistical analyses performed with two-tailed paired Welch's t-test on the mean values of three replicates. **d, e** Same as (**c**) but for single replicates from this study and ref. 3. Lack of replicates precluded calculation of *P* values. Central lines in box plots indicate the median, the box shows the interquartile range, and whiskers indicate data points within 1.5 times of the interquartile range.

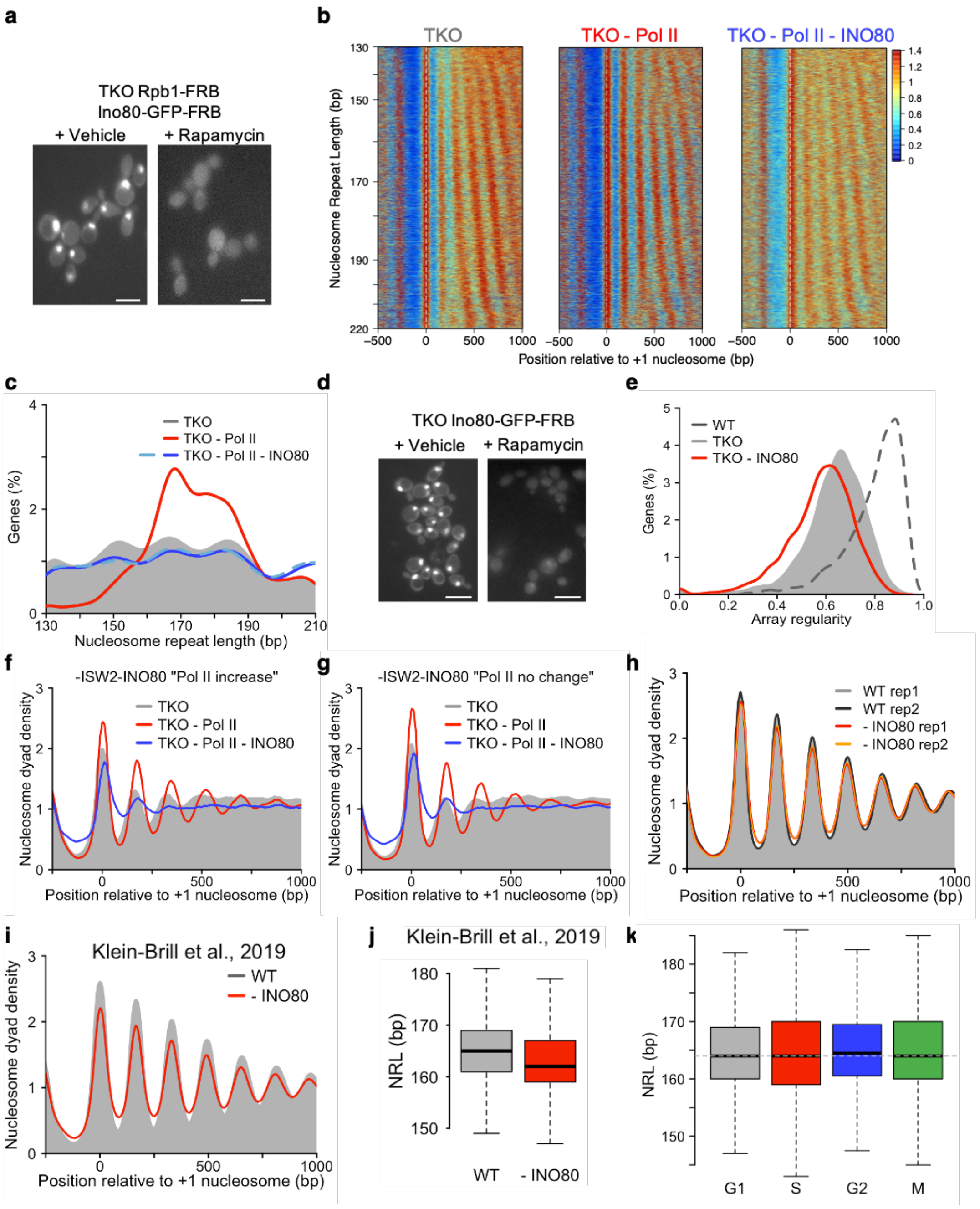

Supplementary Figure 4: The INO80 complex helps generate regular nucleosome arrays. a Live-cell imaging of GFP-FRB-tagged INO80 shows its depletion from the nucleus. Treatment time 2 h. Scale bar 5 µm. **b** Heatmaps showing nucleosome organization upon Pol II- or combined Pol II- and INO80-depletion for 2 h in TKO cells. The TKO control is a 2 h vehicle-treated Rpb1-FRB TKO strain. Genes are sorted by NRL measured in the TKO control. **c** NRL distribution of samples in **b**. **d** Live cell imaging for TKO Ino80-GFP-FRB cells. Treatment time 1.5 h. Images in (a, d) are representative of two biological replicates. Scale bar 5 µm. **e** Array regularity distribution upon INO80 depletion in TKO cells. **f** Average nucleosome organization at 1592 genes that experience increased Pol II occupancy upon ISW2 and INO80 depletion, as identified in ref. 4. **g** Same as **f**, but for genes (3330) that do not show increased Pol II levels. **h, i** Nucleosome organization upon INO80 depletion in WT cells (**h**, anchor-away; **i**, auxin-induced degradation from ref. 5). **j,** NRL distribution upon INO80 depletion in WT cells. Data from ref. 5. **k,** NRL distribution during G1, S, G2 and M cell cycle phases in WT cells. Data from ref. 6. Horizontal line is at 164 bp. Central lines in box plots indicate the median, the box shows the interquartile range, and whiskers indicate data points within 1.5 times of the interquartile range.

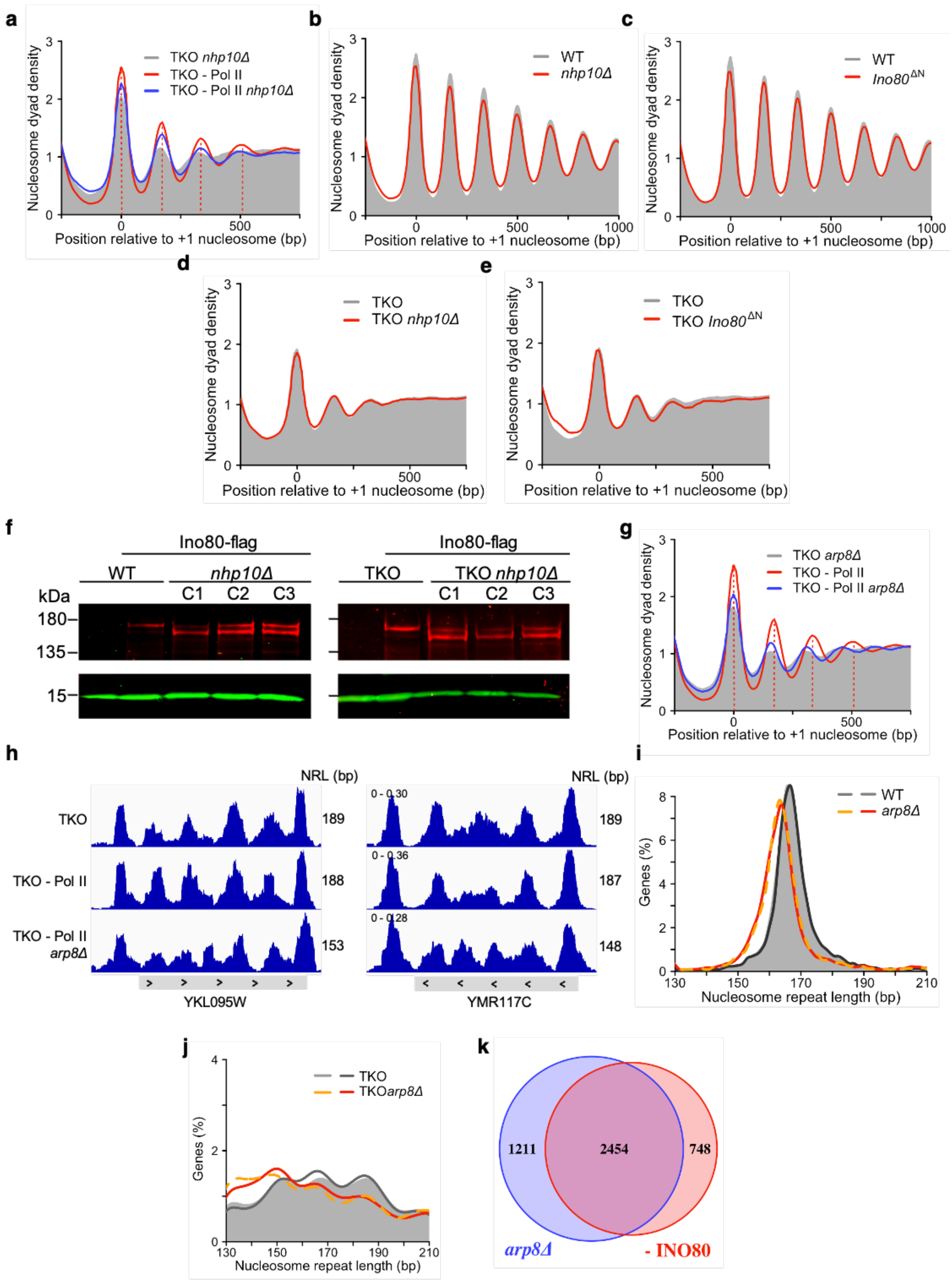

Supplementary Figure 5: Role of INO80 domains and subunits in nucleosome organization. a Deletion of *NHP10* does not alter the position of nucleosome peaks that arise upon Pol II depletion (1 h) in TKO cells (dashed lines). **b** Deletion of *NHP10* from WT cells has negligible effects on global NRL and regularity. Reanalysis of published data[7] confirmed results (not shown). **c** Deletion of 300 N-terminal amino acids of Ino80 does not affect the genome-wide nucleosome organization. **d, e** The nucleosome organization remains similar upon deletion of *NHP10* or Ino80's N-terminus in TKO cells.

f Western blots showing N-terminal degradation of the Ino80 ATPase upon *NHP10* deletion in WT and TKO backgrounds. The Ino80 ATPase was C-terminally FLAG-tagged where indicated and anti-FLAG M2 antibody was used. Three independent *nhp10Δ* clones (C1, C2 and C3) were tested. Source data are provided as a Source Data file. **g** Same as **a**, but for *arp8Δ*. The NRL visibly shortened upon *arp8Δ* deletion. **h** IGV browser view of the indicated strains. *ARP8* deletion shortened the nucleosome-to-nucleosome distance. **i** NRL distribution in WT and *arp8Δ* cells. Peak maxima are at 166 bp and 163 bp, respectively. **j** Deletion of *ARP8* shifts the NRL distribution to lower values in TKO cells. **k** Venn diagram of genes that experience a decreased NRL upon INO80 depletion or *ARP8* deletion in otherwise WT cells.

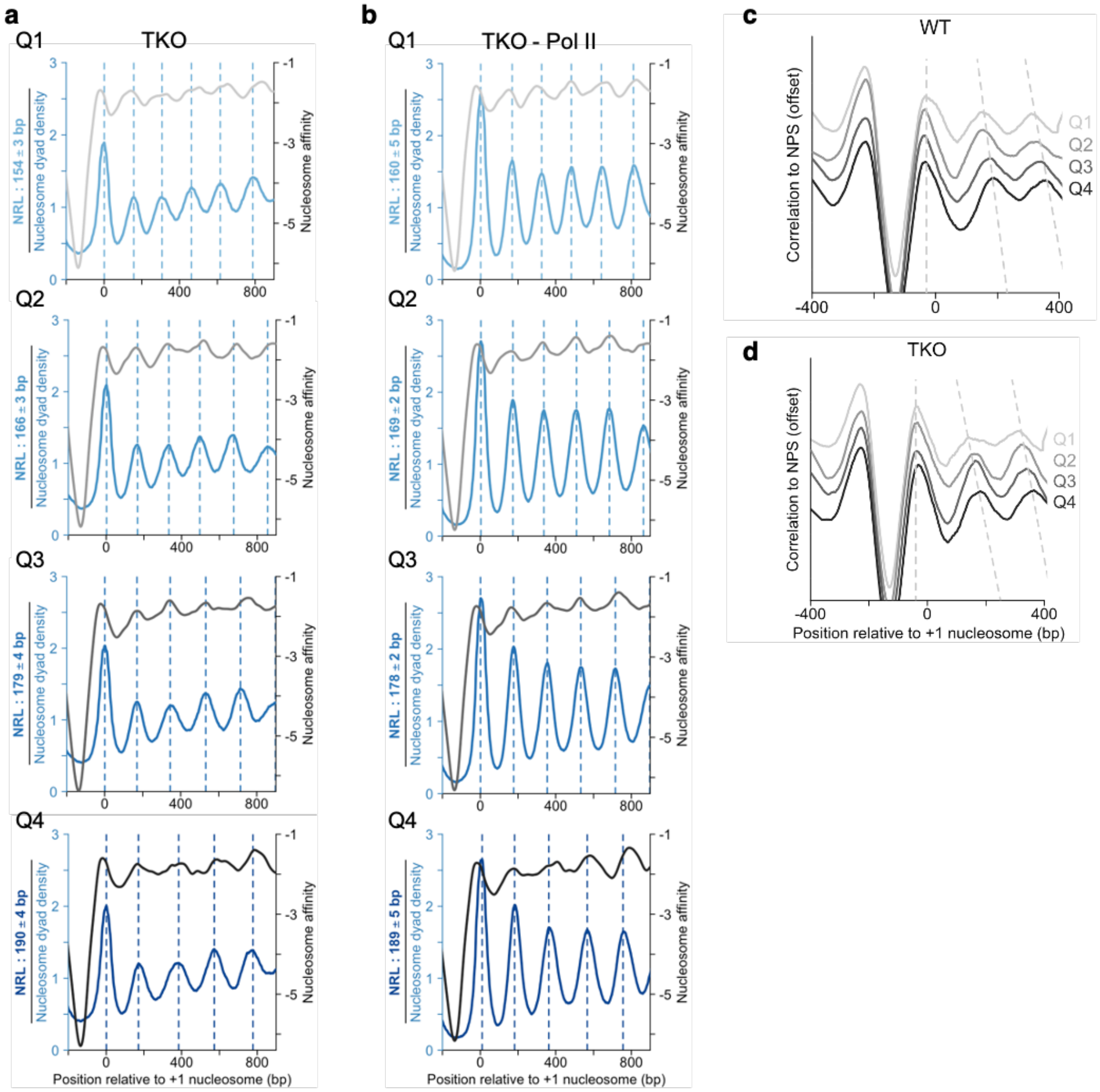

Supplementary Figure 6: DNA sequence influences the NRL over genes. a Comparison of MNase-seq data from TKO cells with predicted nucleosome affinities. Genes were divided into quartiles according to their experimentally determined NRL. Mean values and SD of NRLs are provided on the left of each quartile. **b** Same as **a**, but for Pol II-depleted TKO cells. **c, d** An alternative nucleosome prediction algorithm[8] (Nucleosome Positioning Sequence or NPS) reproduces results obtained in **Figures 6b,c** respectively.

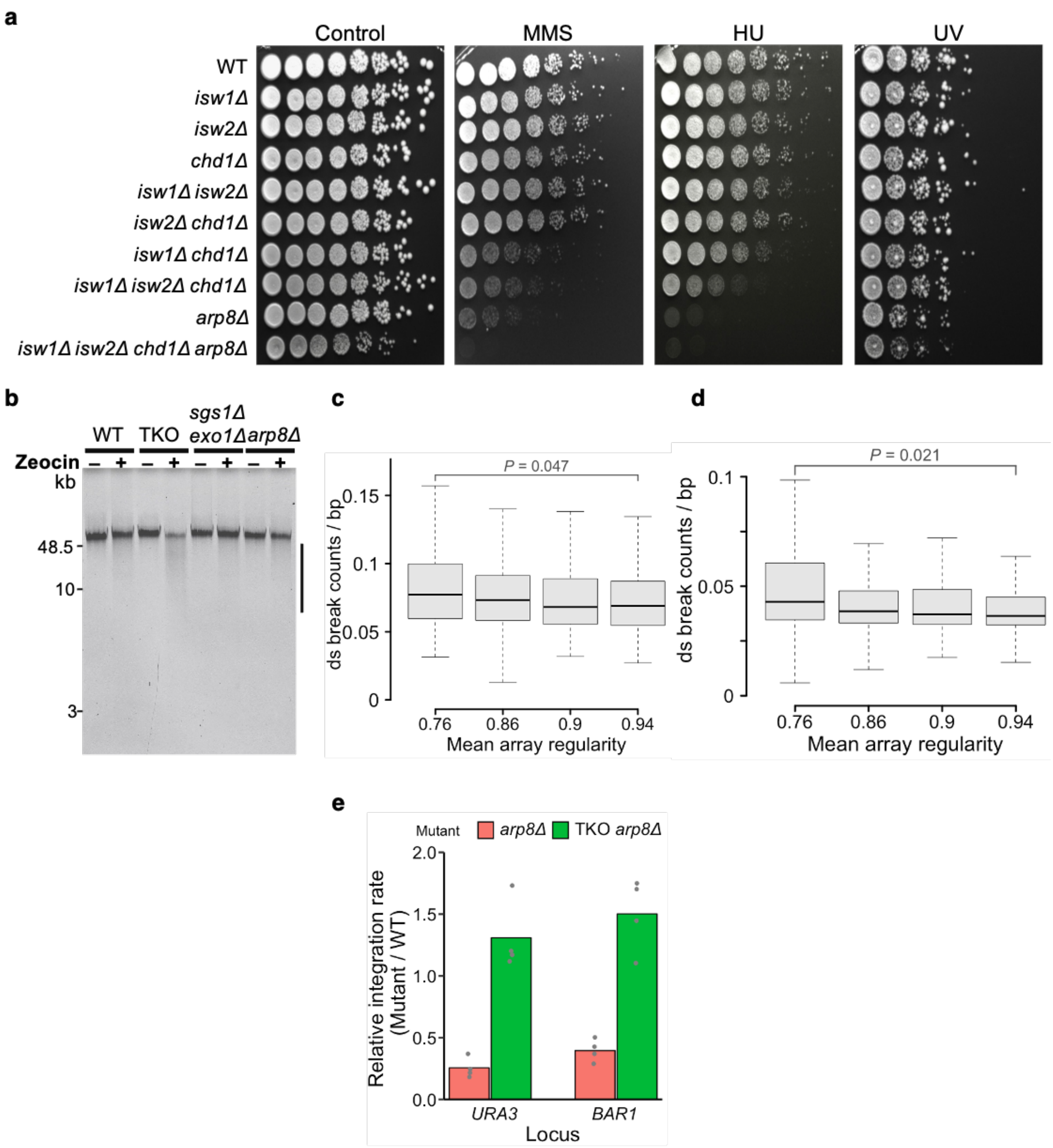

Supplementary Figure 7: Array regularity affects the function of the genome. a Growth assay for indicated yeast strains on YPAD upon Methyl methanesulfonate (MMS; 0.05%), Hydroxyurea (HU; 200 mM) or UV–treatment (200 J/m^2). **b** Replicate from **Figure 7b**. **c** Topoisomerase 2-induced DNA ds breaks that naturally occur during meiosis[9] enrich in genes with low array regularity. **d** Spo11-induced DNA ds breaks[9] enrich in genes with low array regularity. *P* values in (**c**, **d**) represent statistical analyses performed with two-tailed paired Welch's t-test on the mean values of two replicates. **e** Homologous recombination tested at two genomic loci (*URA3*, *BAR1*) in indicated mutants. Dots indicate four individual replicates. Source data are provided as a Source Data file. Central lines in box plots indicate the median, the box shows the interquartile range, and whiskers indicate data points within 1.5 times of the interquartile range.

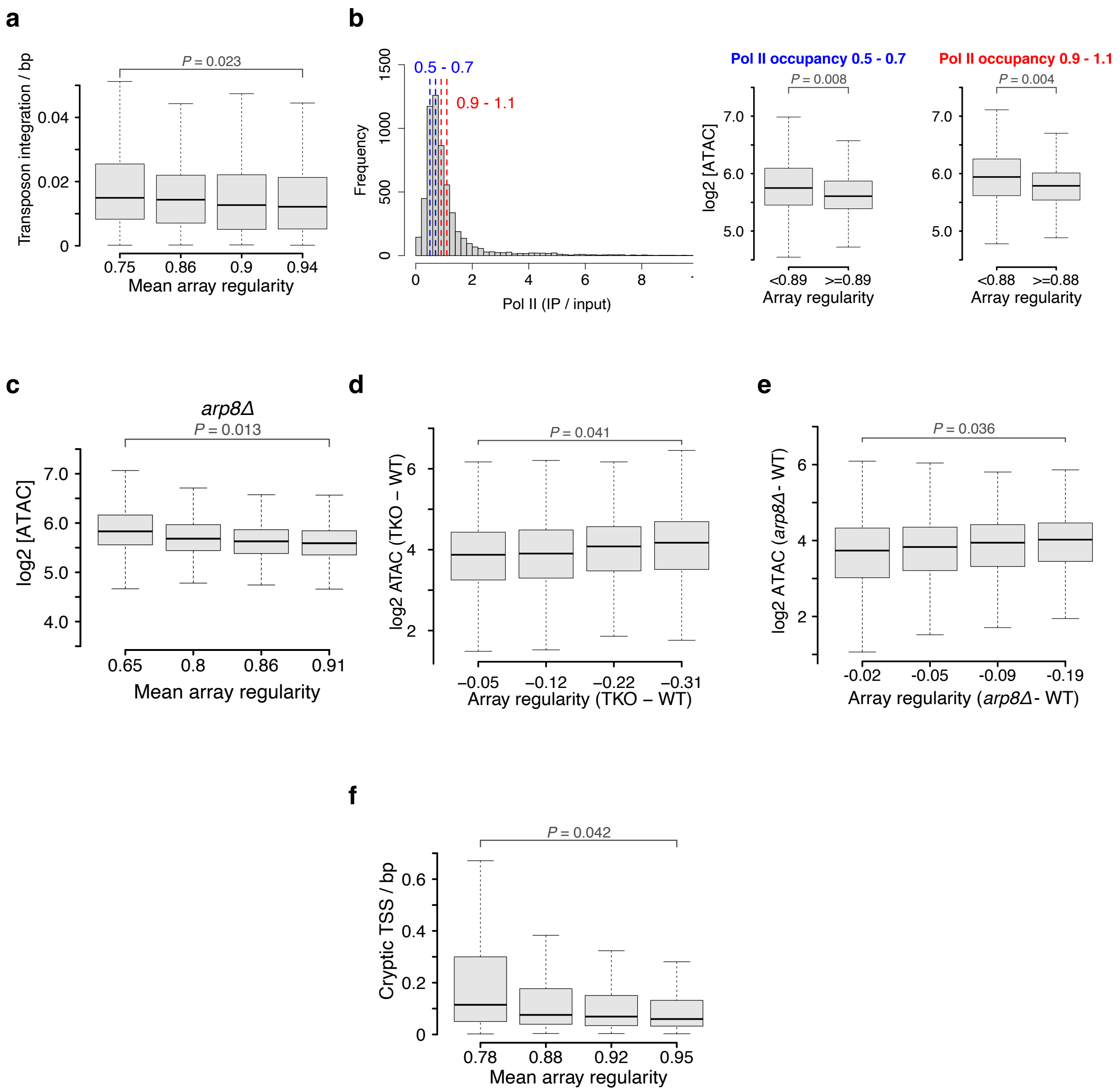

Supplementary Figure 8: Array regularity limits chromatin accessibility. a Ectopically induced transpositions *in vivo* anti-correlate with array regularity. Transposition data is from ref. 10. **b** Low nucleosome array regularity associates with high ATAC-Seq insertion frequencies in WT cells for genes selected to have similar transcription strengths. From the distribution of the RNA Pol II signal[11] (left panel), we selected genes with a signal either between 0.5 and 0.7 (middle panel; 1296 genes) or 0.9 and 1.1 (right panel; 708 genes). These genes were further divided into two groups based on their median array regularity, as indicated. **c** The number of ATAC-seq insertions into gene bodies anti-correlates with array regularity in *arp8Δ* cells. **d, e** Gene quartiles that experience the largest drop in array regularity upon introduction of the TKO or *ARP8* mutation gain more ATAC-seq signal over gene bodies. **f** Cryptic TSSs are enriched in gene bodies with low array regularities. TSS data from ref. 12. *P* values represent statistical analyses performed with two-tailed paired Welch's t-test on the mean values of two replicates. Central lines in box plots indicate the median, the box shows the interquartile range, and whiskers indicate data points within 1.5 times of the interquartile range.

Supplementary Table 1. Plasmids used.

List of all plasmids used in this study.

Plasmid number	Description
pFMP519	pRS413 Gal1-10 *HHT2-HHF2*
pFMP549	pRS416 ARP8
pFMP550	pRS416 arp8$^{\Delta N}$

Supplementary References

1. Mann, R. K. & Grunstein, M. Histone H3 N-terminal mutations allow hyperactivation of the yeast GAL1 gene in vivo. *EMBO J.* **11**, 3297–3306 (1992).
2. Tramantano, M. *et al.* Constitutive turnover of histone H2A.Z at yeast promoters requires the preinitiation complex. *eLife* **5**, e14243 (2016).
3. Kubik, S. *et al.* Nucleosome Stability Distinguishes Two Different Promoter Types at All Protein-Coding Genes in Yeast. *Mol. Cell* **60**, 422–434 (2015).
4. Kubik, S. *et al.* Opposing chromatin remodelers control transcription initiation frequency and start site selection. *Nat. Struct. Mol. Biol.* **26**, 744–754 (2019).
5. Klein-Brill, A., Joseph-Strauss, D., Appleboim, A. & Friedman, N. Dynamics of Chromatin and Transcription during Transient Depletion of the RSC Chromatin Remodeling Complex. *Cell Rep.* **26**, 279-292.e5 (2019).
6. Deniz, Ö., Flores, O., Aldea, M., Soler-López, M. & Orozco, M. Nucleosome architecture throughout the cell cycle. *Sci. Rep.* **6**, 19729 (2016).
7. Cutler, S., Lee, L. J. & Tsukiyama, T. Chromatin Remodeling Factors Isw2 and Ino80 Regulate Chromatin, Replication, and Copy Number of the *Saccharomyces cerevisiae* Ribosomal DNA Locus. *Genetics* **210**, 1543–1556 (2018).
8. Ioshikhes, I. P., Albert, I., Zanton, S. J. & Pugh, B. F. Nucleosome positions predicted through comparative genomics. *Nat. Genet.* **38**, 1210–1215 (2006).
9. Gittens, W. H. *et al.* A nucleotide resolution map of Top2-linked DNA breaks in the yeast and human genome. *Nat. Commun.* **10**, 4846 (2019).
10. Michel, A. H. *et al.* Functional mapping of yeast genomes by saturated transposition. *eLife* **6**, e23570 (2017).
11. Ocampo, J., Chereji, R. V., Eriksson, P. R. & Clark, D. J. The ISW1 and CHD1 ATP-dependent chromatin remodelers compete to set nucleosome spacing *in vivo*. *Nucleic Acids Res.* **44**, 4625–4635 (2016).
12. Lu, Z. & Lin, Z. Pervasive and dynamic transcription initiation in *Saccharomyces cerevisiae*. *Genome Res.* **29**, 1198–1210 (2019).
