# Supplementary material for: The biogenesis and function of nucleosome arrays
Source: Nat Commun. 2021 Dec 1;12:7011. doi: 10.1038/s41467-021-27285-6 (PMC8636622; doi:10.1038/s41467-021-27285-6)
Supplement: Supplementary file 2 — Description of additional Supplementary File [file 41467_2021_27285_MOESM2_ESM.pdf]

Description of additional Supplementary data files

Supplementary Data 1
Description: This file lists all S. cerevisiae strains used in this study.

Supplementary Data 2. Oligonucleotide
Description: This file lists all primers used in this study.
