# Supplementary material for: The biogenesis and function of nucleosome arrays
Source: Nat Commun. 2021 Dec 1;12:7011. doi: 10.1038/s41467-021-27285-6 (PMC8636622; doi:10.1038/s41467-021-27285-6)

Supplementary Fig 1b left


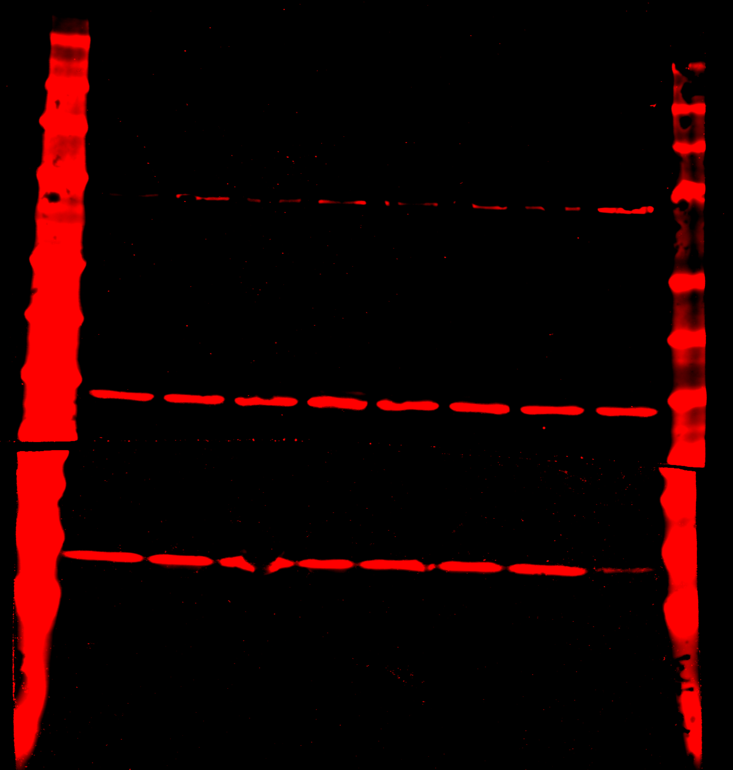


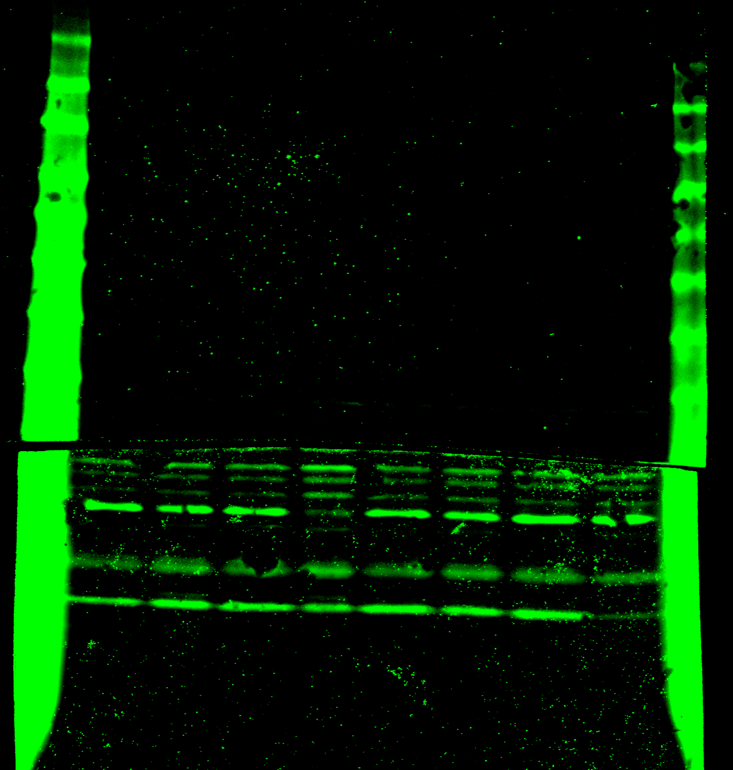


Supplementary Fig 1b right


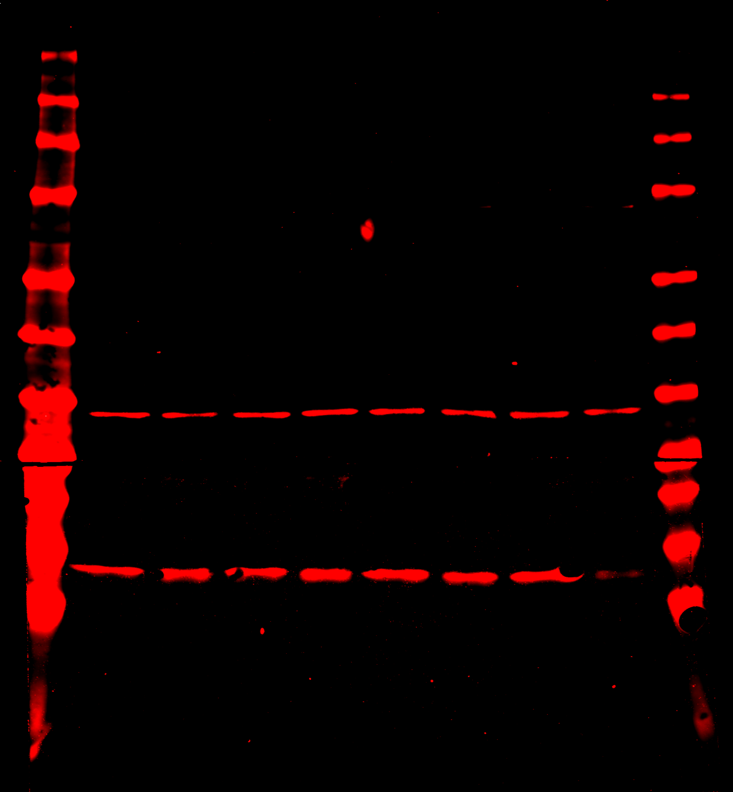


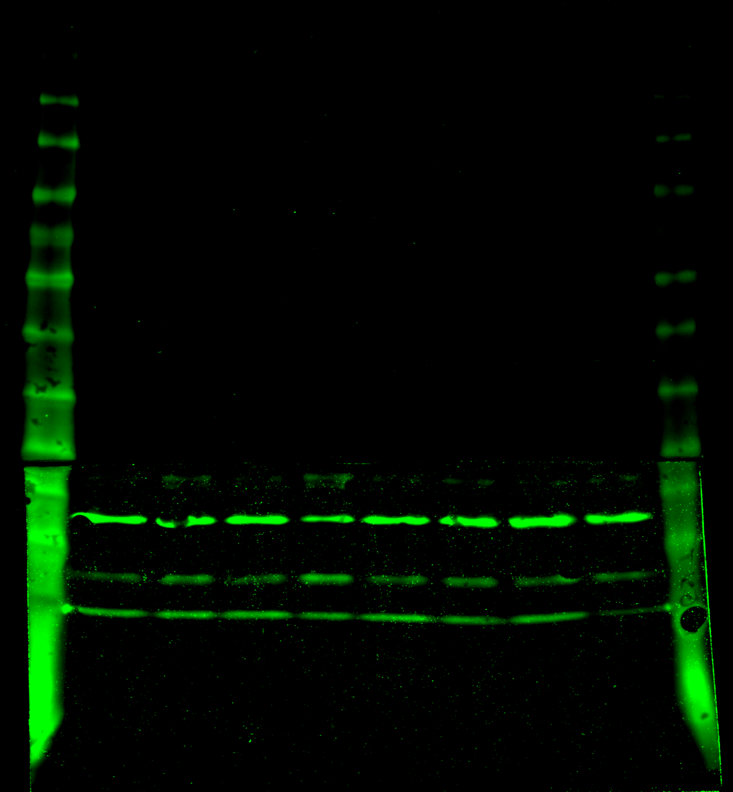


Supplementary Fig 5f left


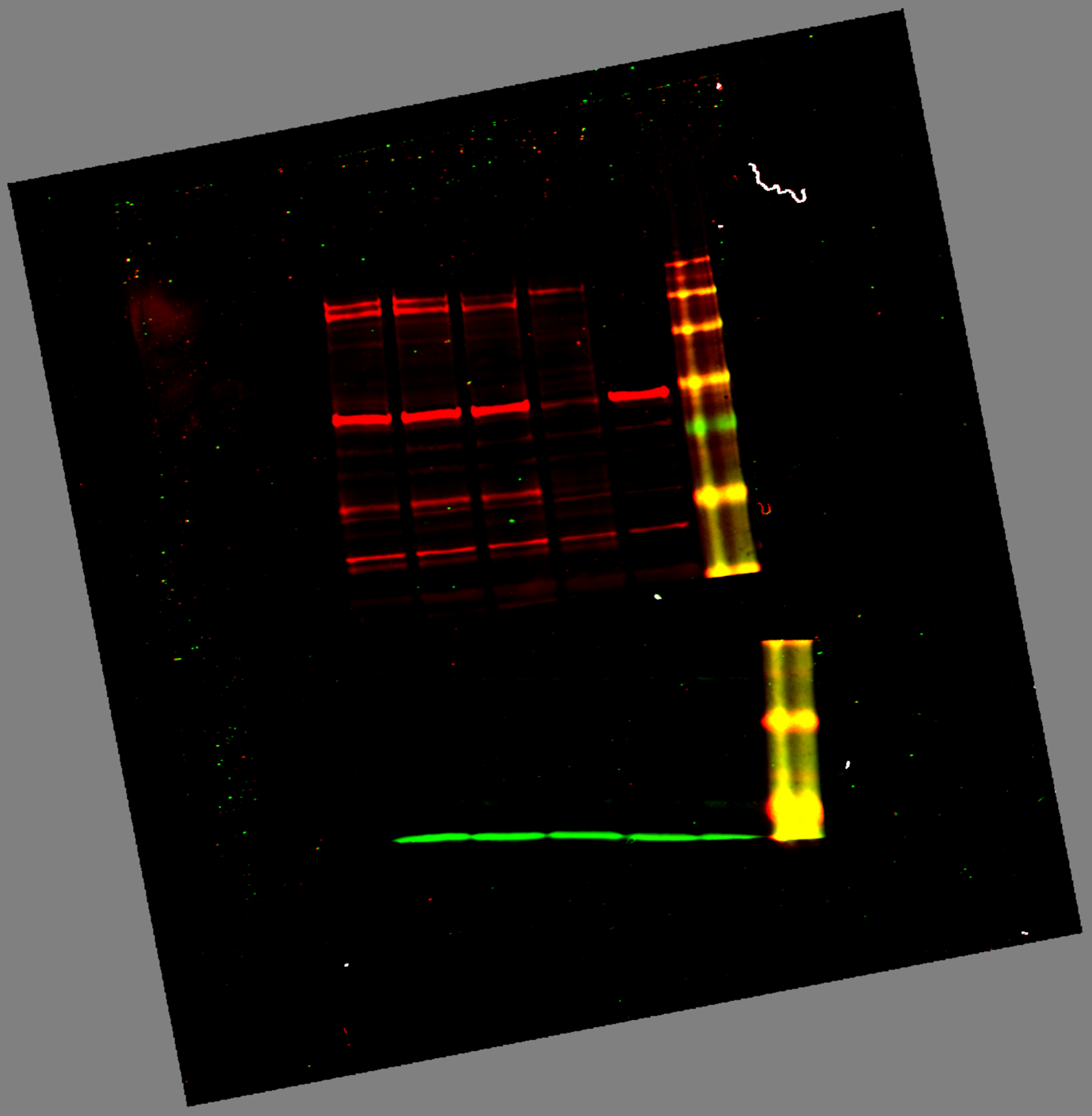


Supplementary Fig 5f right


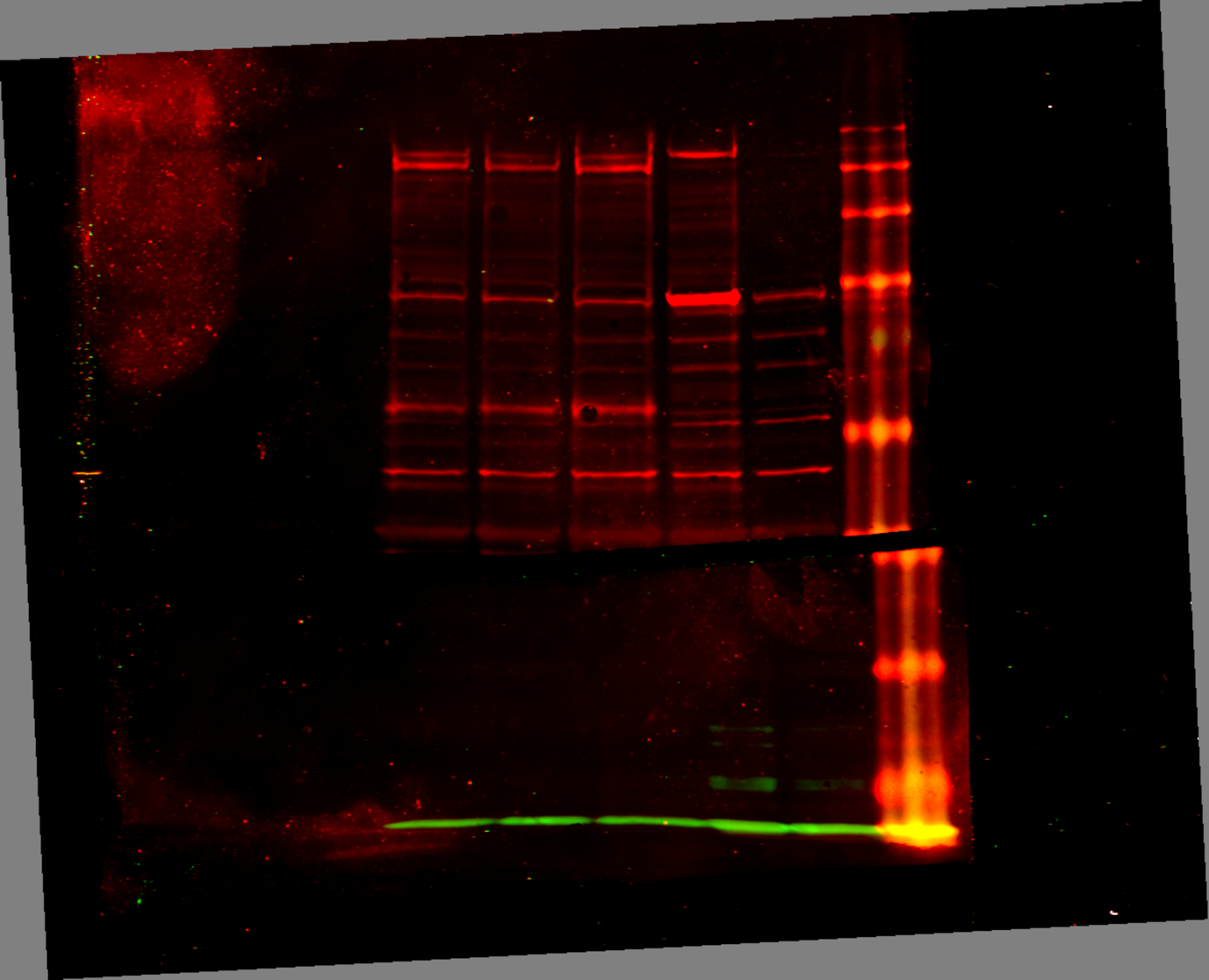


Figure 7b


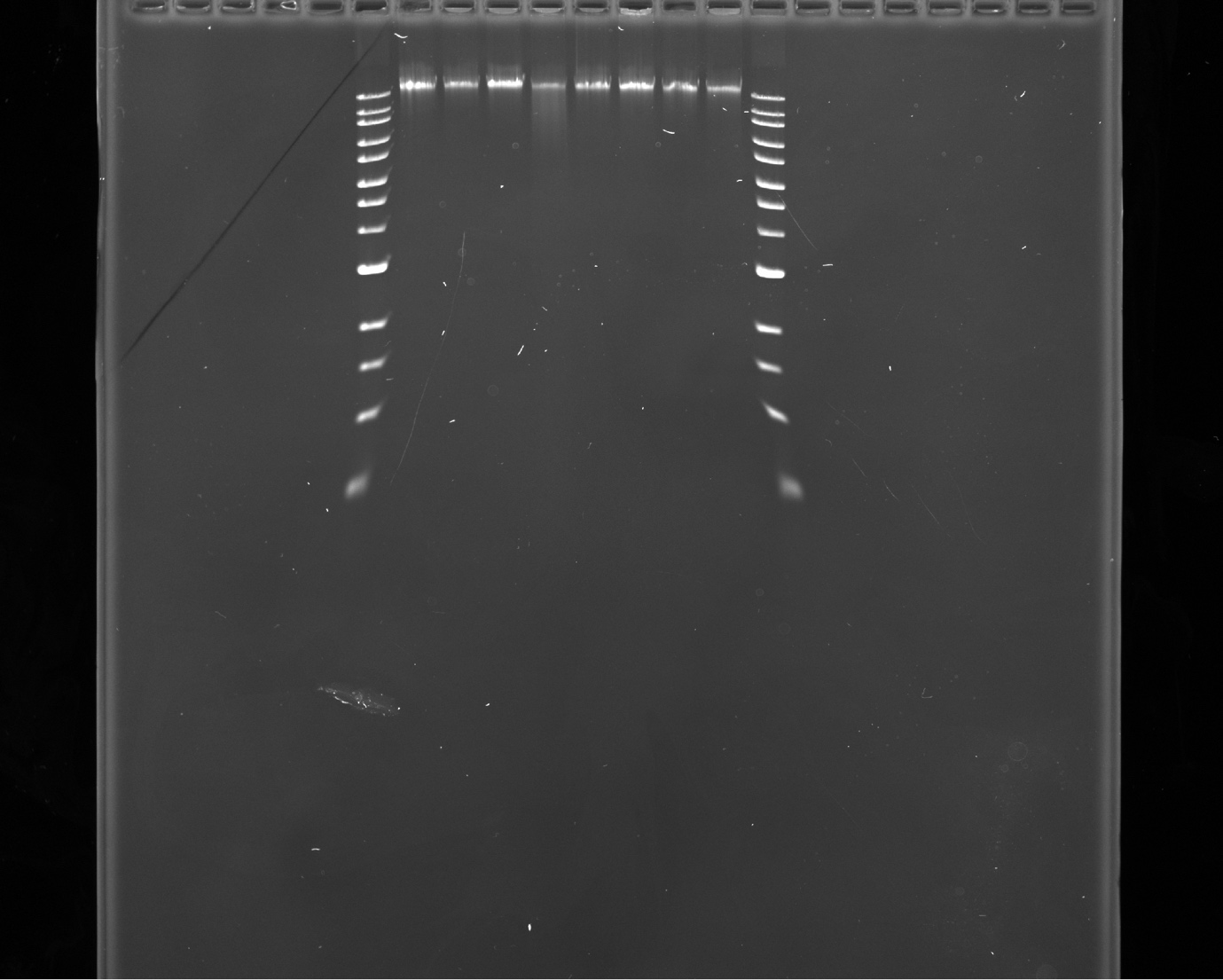


Supplementary Fig 7b


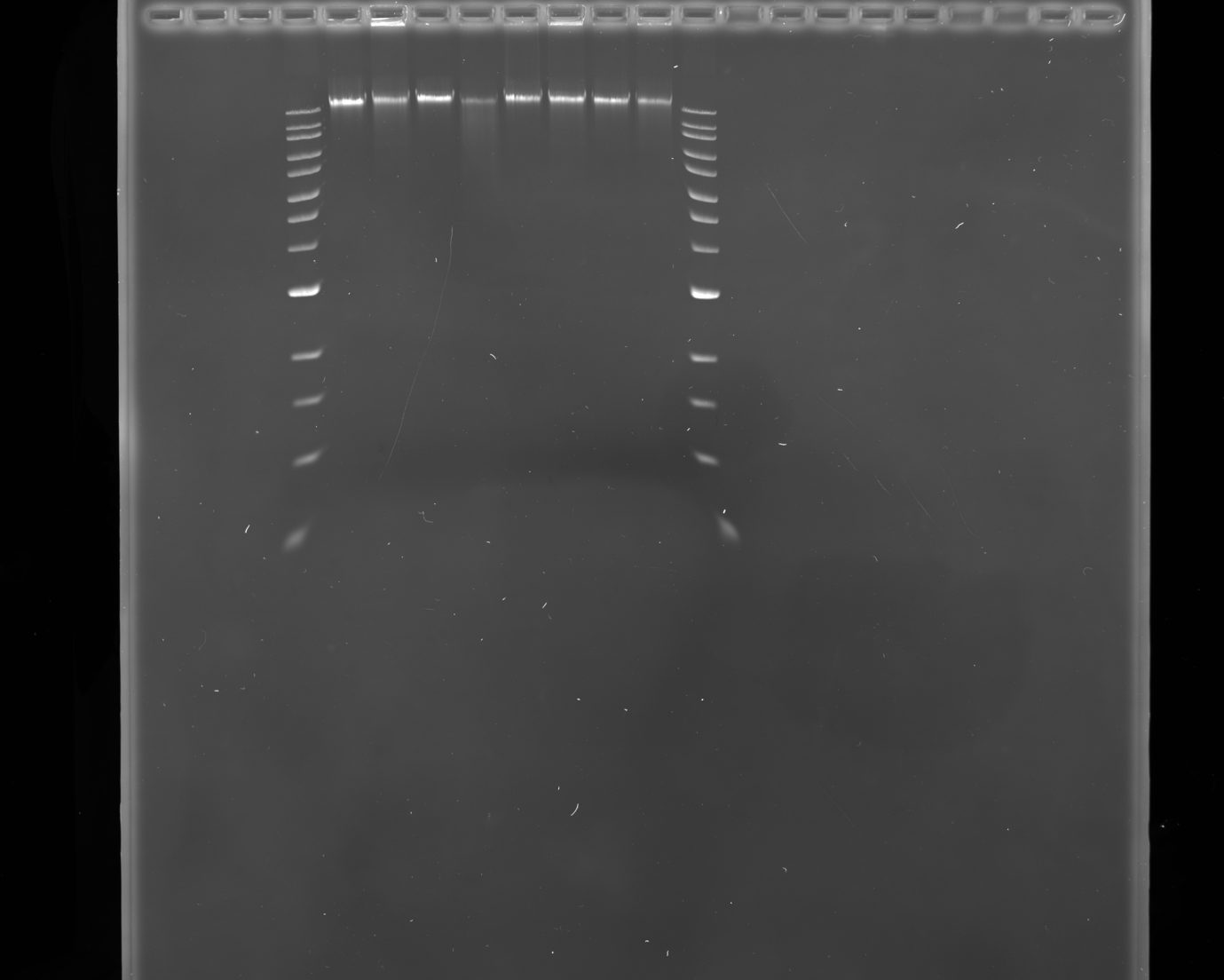

Supplement: Supplementary file 6 — Source Data [file 41467_2021_27285_MOESM6_ESM.zip › Source Data Gels.docx]
